# Viscoelastic Polyurethane Foams with Reduced Flammability and Cytotoxicity

**DOI:** 10.3390/ma15010151

**Published:** 2021-12-26

**Authors:** Małgorzata Okrasa, Milena Leszczyńska, Kamila Sałasińska, Leonard Szczepkowski, Paweł Kozikowski, Adriana Nowak, Justyna Szulc, Agnieszka Adamus-Włodarczyk, Michał Gloc, Katarzyna Majchrzycka, Joanna Ryszkowska

**Affiliations:** 1 Department of Personal Protective Equipment, Central Institute for Labour Protection—National Research Institute, Wierzbowa 48, 90-133 Lodz, Poland; agada@ciop.lodz.pl (A.A.-W.); kamaj@ciop.lodz.pl (K.M.); 2 Faculty of Materials Science and Engineering, Warsaw University of Technology, Wołoska 141, 02-507 Warszawa, Poland; milena.leszczynska.dokt@pw.edu.pl (M.L.); kamila.salasinska@pw.edu.pl (K.S.); michal.gloc.wim@pw.edu.pl (M.G.); joanna.ryszkowska@pw.edu.pl (J.R.); 3 Department of Chemical, Aerosol and Biological Hazards, Central Institute for Labour Protection—National Research Institute, Czerniakowska 16, 00-701 Warszawa, Poland; pakoz@ciop.pl; 4 FAMPUR Adam Przekurat Company, Gersona 40/30, 83-305 Bydgoszcz, Poland; leonardosz@interia.pl; 5 Department of Environmental Biotechnology, Lodz University of Technology, 90-530 Lodz, Poland; adriana.nowak@p.lodz.pl (A.N.); justyna.szulc@p.lodz.pl (J.S.)

**Keywords:** viscoelastic polyurethane foams, respiratory protective devices, customisation, limited flammability, cytotoxicity, leak tightness, comfort

## Abstract

Consistent and proper use of respiratory protective devices (RPD) is one of the essential actions that can be taken to reduce the risk of exposure to airborne hazards, i.e., biological and nonbiological aerosols, vapours, and gases. Proper fit of the facepiece and comfort properties of RPDs play a crucial role in effective protection and acceptance of RPDs by workers. The objective of the present paper was to develop viscoelastic polyurethane foams for use in RPD seals characterised by proper elasticity, allowing for the enhancement of the device fit to the face and the capability of removing moisture from the skin in order to improve the comfort of RPD use. Moreover, it was pivotal to ensure the non-flammability of the foams, as well as a simultaneous reduction in their cytotoxicity. The obtained foams were characterised using scanning electron microscopy, infrared spectroscopy, thermogravimetry, and differential scanning calorimetry. Measurements also involved gel fraction, apparent density, compression set, rebound resilience, wettability, flammability, and cytotoxicity. The results are discussed in the context of the impact of modifications to the foam formulation (i.e., flame-retardant type and content) on the desired foam properties. The test results set directions for future works aimed to develop viscoelastic polyurethane foams that could be applied in the design of respiratory protective devices.

## 1. Introduction

Extensive research has recently been carried out to improve the fit of respiratory protective devices (RPDs) to the user’s face and hence increase the comfort of use [1,2,3,4,5]. To that end, viscoelastic polyurethane (PUR) foams were used as sealing materials in the RPD facepieces [6]. In addition to the improved fit to the body shape, they remove heat from the skin surface and reduce pressure, hence stimulating blood circulation and increasing user comfort. The described effect has already been observed in household objects, such as pillows, seats, and mattresses [7,8]. For materials intended for respiratory protective devices, it is indispensable to ensure some additional functional characteristics resulting from the specific use conditions and reference standards harmonised with Regulation No. (EU) 2016/425 of the European Parliament and the Council on personal protective equipment (PPE) [9], including but not limited to reduced susceptibility to ignition, capability of removing moisture from the skin surface, and harmlessness in the intended conditions of use. Human factors (i.e., theory, principles, data, and methods concerning the interactions between humans and PPE that apply to design) shall also be considered when designing such devices to optimize human well-being and overall system performance [10,11,12].

From the point of view of seal functionality, a continuous and spontaneous adaptation of shape to the user’s anthropometric dimensions should be possible in order to eliminate any potential leakage when the user is unaware of the danger or is not able to adjust the RPD’s fit because of the nature of the tasks performed (e.g., during rescue or fire-fighting actions). Seals should be flame-resistant, especially for equipment used by fire-fighters and rescue services [13]. Furthermore, seals should be resistant to mechanical factors (e.g., putting it on and taking off repeatedly, fine scratches formed during use, facial hair), absorb or remove liquid from the skin surface, and should not intrinsically pose any hazard (e.g., be toxic for the skin) [14].

Obtaining a material that fulfils all of the requirements mentioned above poses a significant technological challenge for the modification of foam formulation. Polyurethane systems intended for viscoelastic foams are highly sensitive to raw-material system modifications, which can lead to shrinkage of the end product, for example [8]. Compared to standard elastic foams, the low water content in the polyol masterbatch significantly affects the foaming rate balance versus the rate of urethane-urea bond formation. In addition, the type and share of surfactants influence the cellular structure and the share of open cells in the produced material [15].

Limiting flammability of PUR foams, required for materials used in RPD design, while maintaining desired physical, chemical, and functional characteristics is among the challenges related to modification of such foams. Because of their porous structure, PUR foams are highly flammable, which can sometimes limit their application [8]. The ignition point of rigid PUR foam is 310 °C, and its self-ignition point is 415 °C, while thermal resistance in the case of short- and long-term exposure amounts only to 120 °C and 80–100 °C, respectively [8]. Insufficient oxidative stability, which deteriorates physical characteristics and causes material discolouration due to oxidation at a higher temperature, poses a challenge for manufacturers of polyurethane products. Elastic segments are more susceptible to oxidative degradation. In viscoelastic foams, material flammability can be additionally increased by a significant share of open pores, limited water content as a porophore favouring flammable substances (e.g., pentane), and a relatively low isocyanate index.

Different procedures are used to increase thermal stability, delay ignition, and improve fire resistance of polyurethanes. These procedures include the introduction of flame retardants (FR) at the component-mixing stage in the production of polyurethane systems; introduction of compounds containing functional groups, including but not limited to hydroxyl groups, which are subject to chemical bonding with polymer chains; and covering the foam surface with flame retardants [16,17,18,19]. The effects of the dimensions of graphite oxide nanoparticles on the layer-by-layer self-assembly and flame-retardant properties of GO-based coatings deposited on flexible PUR foams have been demonstrated in the literature as a promising route to improve the fire safety of such materials. Cone calorimetry tests showed that by using the investigated additives, heat-release rates could be reduced by approx. 50%, and total smoke-release by approx. 70% [20]. Desirable fire resistance was also shown with microporous poly(vinyl alcohol) (PVA) aerogel/silica, prepared by growing a conformal silica coating onto a PVA aerogel scaffold since the layer served as a barrier for heat transfer and mass loss of the coated organic materials [21].

Although the application of flame retardants improves the fire resistance and thermal stability of PUR foams, it can also lead to technological problems and deteriorate the physical and mechanical characteristics, as well as dimensional stability, of the end products [8]. In particular, it can increase cytotoxicity, which is undesirable for products that come into contact with human tissues [22]. That is why this paper aimed to carry out a series of technological experiments related to the production of viscoelastic PUR foams with physical and chemical characteristics that enable their application as facepiece seals in respiratory protective devices, taking into account the expected conditions of equipment use and the safety and comfort of users.

## 2. Materials and Methods

### 2.1. Viscoelastic Polyurethane Foams

A series of 7 viscoelastic polyurethane foams were prepared using the two-component system based on Fampur (Bydgoszcz, Poland) formulation, at 1.2 php (parts per hundred parts of polyol) water content and 0.9 isocyanate index. Polyol masterbatches (component A) were prepared by placing catalysts, surfactant, polyols, and water (proprietary formulation by Fampur company), weighed with an accuracy of 0.1 g (WTC 300, Radwag, Radom, Poland), in 500 mL disposable polypropylene containers (Paccor, Skierniewice, Poland). The substrates were homogenised using a high-speed stirrer (Siemens 3 MOT 1LA/080, Siemens, Munich, Germany) at 3000 rpm over 120 s. Flame-retardants characterized in Table 1 were introduced into the polyol masterbatch in amounts ranging from 15 to 30 php (Table 2). Ongronat 4040 TR (polymeric MDI; BorsodChem, Kazincbarcik, Hungary) containing 32.6% of free isocyanate groups at a calculated equivalent value of R_NCO_ = 128.8 was used as the isocyanate component (B). After adding the isocyanate component to the masterbatch, the system was mixed using a high-speed stirrer at 3000 rpm over 6 s.

Dynamic viscosity of the mixtures of polyols with flame retardants (Table 1, flame retardant content in relation to the mixture of polyols) was tested at 25 °C using Brookfield viscosity meter model DV-II + Pro (Brookfield Engineering Laboratories, Middleboro, MA, USA). The systems were mixed for 30 s at 2000 rpm. The mixtures were analysed using an SC4-29 type measuring spindle.

The mixture was poured into a 200 mm × 200 mm × 16 mm mould. After the mould was closed, further foaming was carried out when the mould was positioned at a 20 degrees angle, and the rise was observed through a 2 mm diameter inspection hole located at the top of the mould cover. After filling the inspection hole or a minor outflow (0.5–5 g), the mould was maintained in the above conditions for 6.5–18.5 min. The foam obtained at the open outlet and minor outflow of the mixture was described as the zero value of the overload rate, *P* = 0%. In order to obtain moulded foams with an overload rate of *P* > 0%, the hole was closed. The overload rate was determined based on the formula:

$$P=\frac{M_{1}-M_{0}}{M_{0}}\times 100\%$$

where: M_0_ is the form weight with the open gas-venting outlet and M_1_ is the form weight with the closed gas-venting outlet.

Before removing from the mould, the foams were cured at 70 °C for 30 min and conditioned at ambient temperature for 22 h. Before testing, the samples were conditioned at ambient temperature for 14 days.

### 2.2. Apparent Density

The apparent density of the foams was determined according to the ISO 845 standard [23]. Dimensions of rectangular samples were measured with 0.1 mm accuracy and weighed in the air with an accuracy of 0.001 g using a WPA 180/C/1 scale (Radwag, Radom, Poland).

### 2.3. Microstructure and Porosity

Samples were observed using a Hitachi SU8010 scanning electron microscope (SEM, Hitachi High-Technology Corporation, Tokyo, Japan) following gold sputtering with a Q150T ES device (Quorum Technologies, Lewes, UK). Imaging was performed with secondary electrons at an acceleration voltage of 10 kV and a working distance of 30 mm. The mean equivalent diameters and aspect ratio of pores (*N* ≥ 500 for each foam) were established based on the SEM images.

A XRADIA 410C microtomograph (Carl Zeiss Carl Zeiss, Jena, Germany) with a computer set for image reconstruction was used to visualise the structure of the analysed samples. Each sample intended for porosity analysis was cut to ~10 mm × 10 mm. Then, it was fixed vertically to the table inside the device chamber. The accelerating voltage and power of the beam used in the target test amounted to 80 kV and 10 W, respectively, and the exposure time in a single projection was 3 s. A total of 1441 photos were taken per 180° revolution. The applied filter was marked as #LE1. The scanning time for each sample was 6 h. The position of the phase-contrast-amplifying detector was used. The embedded X-Radia reconstructor programme (Carl Zeiss Carl Zeiss, Jena, Germany) was used to reconstruct the obtained images (slices). The resolution amounted to ca. 18 µm. As a result of the photo processing, 8-bit images were obtained, which enabled the development of a 3D tomographic model of the test sample. The test revealed a volumetric share of porosity in the samples. The images were reconstructed in NRecon (Microphotonics Inc., Allentown, PA, USA). A 2D visualisation of the samples was developed with DataViewer (Microphotonics Inc., Allentown, PA, USA), while the 3D visualisation with was developed with CT Vol (Microphotonics Inc., Allentown, PA, USA). The images obtained after reconstruction were used in CTAn (Microphotonics Inc., Allentown, PA, USA) for quantitative analysis of the microstructure. Binary image development and removal of defects enabled estimation of porosity percentage. The resolution of the method amounted to 8.81 µm.

### 2.4. Surface Wettability

The wettability (contact angle) of the tested surfaces was evaluated using a droplet-deposition method on a Phoenix—Alpha apparatus (SEO, Suwon, South Korea). A droplet volume of 10^−3^ cm^3^ was applied with a syringe onto the sample surface, and photos taken with the camera embedded in the device were analysed with Phoenix Alpha Contact Angle Analyzer software (SEO, Suwon, South Korea). Ten droplets of each test liquid (deionised water, acidic sweat, alkaline sweat, and diiodomethane (Sigma-Aldrich, St. Louis, MO, USA) were placed on each sample. The contact-angle measurements were carried out at room temperature and 37 °C. Before measurement, the samples were conditioned at the referenced temperature for 120 min. The contact angles determined at room temperature were used to calculate the free surface energy. Benchmark fluids with different surface energy—diiodomethane (non-polar liquid L = 50.8 mJ/m^2^, γLd = 50.8 mJ/m^2^ and γLp = 0 mJ/m^2^) and water (bipolar liquid L = 72.8 mJ/m^2^, γLd = 21.8 mJ/m^2^ and γLp = 51 mJ/m^2^)—were used for this purpose. Free surface energy (γ_s_), being the sum of dispersive ($\gamma_{s}^{d}$) and non-dispersive interactions, mainly polar interactions ($\gamma_{s}^{p}$), was determined with the Owens–Wendt method [24].

### 2.5. Fourier Transform Infrared Spectroscopy

A Nicolet 6700 spectrophotometer (Thermo Electron Corporation, Waltham, MA, USA) with an attenuated total reflection (ATR) was used to record the absorption spectra of the foams. Fourier transform infrared spectroscopy (FTIR) analysis was performed for the foam core and skin. The spectra were collected in ATR mode and treated using normalized absorbance. A total of 64 scans in the wavenumber range of 4000–400 cm^−1^ were performed for each foam sample. The results were analysed using Omnic 8.2.0 software (2018, Thermo Fisher Scientific Inc., Waltham, MA, USA).

### 2.6. Differential Scanning Calorimetry

Differential scanning calorimetry (DSC) analysis was carried out using a DSC Q1000 device (TA Instruments, New Castle, DE, USA) to determine the temperature and thermal effects of phase changes. Foam samples weighing 5.0 ± 0.2 mg were placed in sealed aluminium crucibles, cooled down to −90 °C, heated to 220 °C (10 °C/min; first heating cycle), cooled down again to −90 °C (5 °C/min rate), and finally reheated to 220 °C (10 °C/min rate; second heating cycle). The results were analysed using Universal Analysis 2000 ver. 4.5A software (TA Instruments, New Castle, DE, USA).

### 2.7. Thermogravimetric Analysis

Thermogravimetric analysis (TGA) was performed using a TGA Q500 device (TA Instruments, New Castle, DE, USA). Samples weighing 10.0 ± 0.5 mg were tested under a nitrogen atmosphere upon heating from ambient temperature to 900 °C (10 °C/min rate). The obtained data were analysed using Universal Analysis 2000 ver. 4.7A software (TA Instruments, New Castle, DE, USA).

### 2.8. Compression Set and Rebound Resilience

The compression set was determined according to ISO 1856 [25]. Compression of 50% and 90% was applied for 22 h at 70 °C to 50 mm × 50 mm × 16 mm samples in a direction parallel to the foam growth direction. Resilience was determined according to ISO 8307 [26]. A steel ball with a diameter of 16 mm was dropped from a height of 500 mm onto a 4-layered sample with a layer dimension of 50 mm × 50 mm × 16 mm (total sample dimensions were 50 mm × 50 mm × 64 mm). The height of the rebound was determined based on slow-motion video analysis.

### 2.9. Burning Behaviour

Measurements with a cone calorimeter (CC, Fire Testing Technology, East Grinstead, UK) were carried out according to ISO 5660-1 and ISO 5660-2 [27,28]. Samples of 100 mm × 100 mm × 16 mm were prepared by wrapping the edges with aluminium foil and covering the bottom with a ceramic blanket. Then, the samples were arranged horizontally against the cone radiator and exposed to heat radiation flux of 35 kW/m^2^. The spark ignition was used to ignite the pyrolysis products. An optical system with a silicon photodiode and helium-neon laser enabled continuous measurement of the smoke’s optical density. Three measurements were conducted for each type of foam variant.

### 2.10. Cell Culture and Cytotoxicity Testing

For cytotoxicity testing, the samples were prepared according to EN ISO 10993-12:2012 [29]. Comminuted polyurethane foam samples weighing 2.0 ± 0.5 g were suspended in 20 mL of complete Dulbecco’s Modified Eagle’s Medium (DMEM) for cell culture (Sigma-Aldrich, St. Louis, MO, USA), mixed, and then extracted for 8 h (160 rpm) at 37 ± 1 °C. Each extract was filtered twice through sterile 0.22 µm syringe filters (Membrane Solutions, Kent, WA, USA).

The study used the normal immortalised human keratinocyte cell line HaCaT (the original material created by Prof. Dr. Petra Boukamp and Dr. Norbert Fusenig [30]) from the 35th passage (Cell Line Service GmbH, Eppelheim, Germany). The cells were selected because keratinocytes constitute almost 95% of the epidermis; these cells are the first to contact synthetic materials such as polyurethane. HaCaT cells were cultured as a monolayer in DMEM with the addition of 10% foetal bovine serum (FBS, Gibco, Thermo Fisher Scientific, Waltham, MA, USA), 2 mM glutaMAX^TM^ (Gibco, Thermo Fisher Scientific, Waltham, MA, USA), 25 mM HEPES (Sigma-Aldrich, St. Louis, MO, USA), 100 μg/mL streptomycin, and 100 IU/mL penicillin mixture (Sigma-Aldrich, St. Louis, MO, USA) for 3–5 days at 37 °C in 5% CO_2_ atmosphere in a Galaxy 48S incubator (New Brunswick, UK). After reaching 80% confluence, the cells were detached with TrypLE^TM^ Express (Gibco, Thermo Fisher Scientific, Waltham, MA, USA) for about 8–10 min, centrifuged (182× *g*, 3–5 min) and decanted, and then fresh DMEM was added. Viability was checked with a trypan blue (Sigma-Aldrich, St. Louis, MO, USA) exclusion test. Cytotoxicity was tested with neutral red uptake (NRU) assay according to ISO 10993-5 [31]. The cells were seeded in 96-well plates at 10,000/well in a complete culture medium, followed by incubation for 24 h at 37 °C in 5% CO_2_. The next day, the culture medium was aspirated, and the following concentrations of the test extracts were added: 0.78, 1.56, 3.125, 6.25, 12.5, 25, 50, and 100%. Three independent experiments were carried out. Each concentration of a given sample was tested in four replicates per experiment for 24 h. The cells in the culture medium were a vehicle; wells containing only the medium constituted blanks (background), and cells incubated with dimethyl sulfoxide (DMSO, Sigma-Aldrich, St. Louis, MO, USA) in concentrations from 0.078 to 10% were a positive control. The plates were incubated for 24 h, the tested preparations were aspirated, and neutral red (NR, Sigma-Aldrich, St. Louis, MO, USA) was added in a 50 μg/mL concentration in phosphate buffer saline (PBS, Sigma-Aldrich, St. Louis, MO, USA). The plates were incubated for a further 3 h; then, the NR solution was aspirated and extracted from the cells with a desorbing solution (1% acetic acid (Merck Life Science, Warsaw, Poland), 50% ethanol (Merck Life Science, Warsaw, Poland), 49% distilled water). Absorbance was measured at 550 nm using a 620 nm reference filter in a TriStar2 LB 942 microplate reader (Berthold Technologies GmbH and Co. KG, Bad Wildbad, Germany).

The result determined IC_50_ values and non-toxic/safe doses/concentrations (IC_0_) of PUR foam extracts against an established cell line (HaCaT). Based on the obtained absorbance measurements, the percentage of cell viability was calculated compared to the control (cells only incubated in the culture medium). The percentage of cell viability was calculated as the quotient of the sample’s mean absorbance to the mean absorbance of the control. IC_50_ values were read from the curves. Microscopic evaluation of morphological changes in the HaCaT cells induced by PUR foam extracts was carried out using a Nikon Ts2 inverted microscope with EMBOSS contrast (Nikon, Tokyo, Japan) and a Subra Full HD Color digital camera (Jenoptik, Dresden, Germany), under magnification of 200×.

Cytotoxicity-assay data were analysed using one-way analysis of variance (ANOVA) using OriginPro 6.1 (2008, Northampton, MA, USA) software. Significant differences between the means were compared using Scheffe’s multiple comparison test at *p* < 0.05.

## 3. Results and Discussion

### 3.1. Viscosity

**Figure 1:**
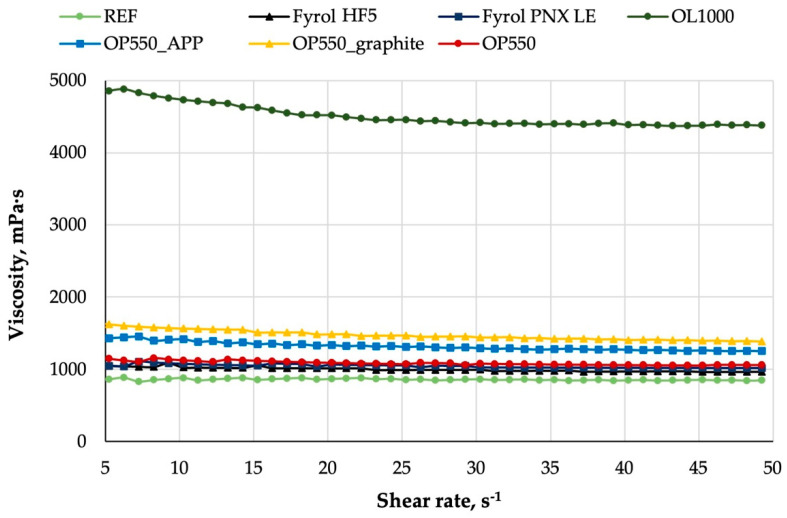
Viscosity curves for a mixture of polyols and polyols with flame retardants.

The viscosity of the analysed mixtures did not depend on shear rate, which indicates that the systems behave like Newtonian fluids (Figure 1).

The introduction of flame retardants to a polyol mixture caused an increase in the viscosity of the systems. Liquid flame retardants, such as Fyrol HF5, Fyrol PNX LE, and OP550, contributed to the increase in viscosity by ca. 200 mPa·s, while the introduction of 15 php of APP fillers and graphite raised the viscosity of the systems by 200–400 mPa·s. The results of viscosity analysis reveal a significant increase in the mixture viscosity after the introduction of 30 php of OL1000 flame retardant. Such high viscosity might cause difficulties in spreading the mixtures in the mould. Furthermore, it could disturb the cellular structure of the obtained foams, deteriorating their properties.

### 3.2. Apparent Density

Analysis results reveal an increase in the apparent density of all materials after the introduction of flame retardants (Table 3). Foams containing Fyrol PNX LE and Fyrol HF5 had a lower apparent density than other foams containing FR. This resulted from a lower FR content (20 php) compared to other materials with FR (30 php). The addition of OP550 and APP at a 15/15 php ratio resulted in a foam with the highest apparent density of all analysed samples.

### 3.3. Microstructure and Porosity

**Figure 2:**
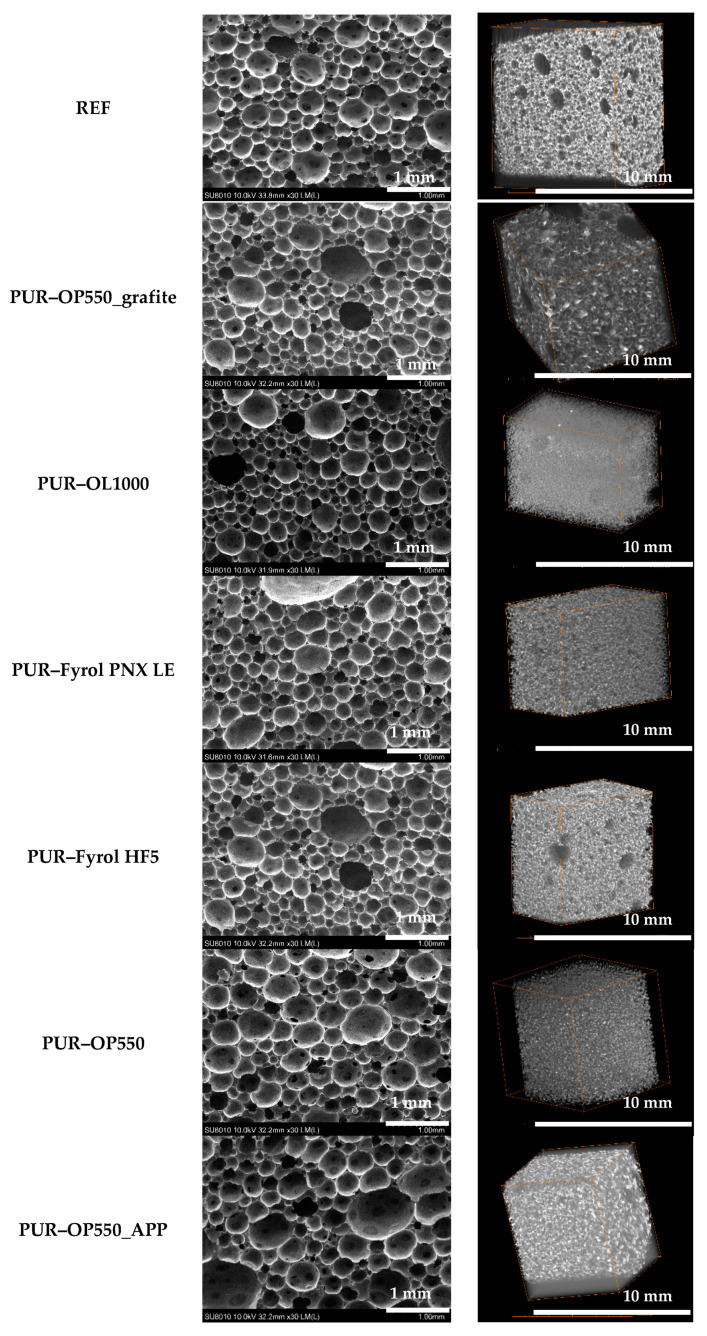
Microstructure of foams by flame retardant.

Figure 2 summarises the microstructure analysis results obtained with SEM (left side) and µCT analysis (right side).

SEM images and the analysis results of the pore dimensions and shape (Table 4) confirm some differences in pore size, depending on the applied flame retardant. The smallest mean diameters of pores were observed for PUR–OL1000 foam, which revealed much higher viscosities than other foams, while the largest pore diameters were obseved for foams containing PUR–OP550_APP. In the case of all types of foam, pores were oval in shape with a similar aspect-ratio value. No agglomerates of flame retardants were observed, which confirms their good dispersion in polyurethane systems.

Processing of images developed with X-ray microtomography resulted in 8-bit images, which enabled attainment of a 3D tomographic model of the test samples (Figure 2). The samples had similar total porosity. The highest values were reported for samples modified with OP550_AAP, and next for OP550 and the reference sample. The test results suggest that the introduction of flame retardants to a polyurethane system does not significantly affect total porosity. Total porosity values obtained with X-ray microtomography correspond to pore-size values obtained with scanning electron microscopy, i.e., the higher the mean pore size, the higher the total porosity.

### 3.4. Surface Wettability

Contact-angle and surface-energy analysis results for foams containing flame retardants are summarised in Table 5.

According to the literature, values of human-skin contact angle with water range from 80 to 110° [32]. In our case, values of foam contact angles were diversified and ranged from 55° (PUR–OP550_APP, distilled water, normal conditions) to 97° (REF, acidic sweat, 37 °C). This means that foam contact angles were within the range of human-skin contact angles. The test results reveal minor differences between the contact angles reported for water and synthetic sweat, measured at different temperatures between the tested foam variants. Most were hydrophilic, which fosters penetration of body fluids into the foam and effective removal of moisture from the skin. The highest contact-angle values were measured for foam that did not contain flame retardants (REF), modified sample PUR–Fyrol HF5, and graphite-modified sample (PUR–OL550_graphite). The lowest contact-angle values were observed for PUR–OP550_APP foams. The highest surface-energy values were reported for PUR–OP550 foams, and the lowest for PUR–Fyrol PNX LE and PUR–OP550_graphite.

Usually, high free surface energy results in good adhesion properties. The highest values of the polar component were reported for the REF and PUR–Fyrol HF5 samples, which is evidence of an increase in surface polarity and stronger interaction of molecular forces between the surfaces, as well as a consequent increase in adhesive strength. PUR–OP550_APP foams achieved the lowest values. When the surface-energy value of a material is high, it can confirm high chemical reactivity and a consequent susceptibility to hydrolysis. The PUR–OP550_APP sample revealed the highest value of the dispersive component.

### 3.5. Fourier Transform Infrared Spectroscopy

**Figure 3:**
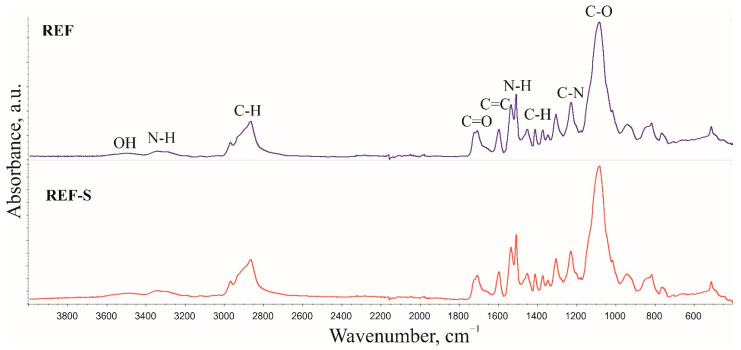
Fourier transform infrared spectroscopy spectra of the reference sample.

FTIR analysis was performed for the foam core and skin. The spectra were collected in ATR mode and treated using normalized absorbance. The FTIR spectra of the reference sample are shown in Figure 3. Samples cut from the foam skin were additionally marked with the letter “S”.

Band characteristics of polyurethanes were observed in the FTIR spectra, suggesting the correct course of the synthesis. A broad band in the wavenumber range of 3600–3400 cm^−1^ comes from stretching vibrations of –OH groups from polyol hydroxy groups; their presence results from the application of an isocyanate index lower than 1. A broad peak is observed in the 3400–3200 cm^−1^ range. This results from asymmetrical and symmetrical stretching vibrations of the –N–H group in urethane domains and urea derivatives. Bands with a maximum at wavenumber values of 1536 cm^−1^ and 1510–1509 cm^−1^ suggest deformation vibrations of the group. The bands at the wavenumbers 2969–2967 cm^−1^ and 2867 cm^−1^ come from asymmetrical and symmetrical stretching vibrations of the –CH group from the CH_3_ and CH_2_ domains. Bands with maximums at 1453 cm^−1^ (CH_3_) and 1373 cm^−1^ (CH_2_) resulting from asymmetrical and symmetrical deformation angles and the band with a maximum at 1306 cm^−1^ coming from stretching vibrations also originate from these groups. The multiplet signals in the 1770–1640 cm^−1^ range confirm the presence of C=O carbonyl groups in the urethane and urea domains. The signal values of 1597 cm^−1^ originate from the C=C group’s stretching vibrations in the aromatic ring, while the band for with a wavenumber of 1230 cm^−1^ comes from the C–N group’s stretching vibrations. The signal from the maximum at a wavenumber of 1086 cm^−1^ comes from the C–O group stretching vibrations, which form polyurethane elastic segments. No signal with a maximum at 2270 cm^−1^ was observed in the FTIR spectra of the foams, which suggests a complete reaction of the NCO group [8,33,34,35,36].

Additional signals related to the introduction of flame retardants were observed in foams modified with such agents.

Figure S1 (Supplementary Material) summarises the FTIR spectra of the reference foam, foam modified with OL1000 (PUR–OL1000) flame retardant and OL1000 additive. For the foam modified with OL1000, additional signals were observed in the wavenumber range of 1280–1150 cm^−1^ and for values under 1000 cm^−1^ (out-of-plane area—the range of –H–Csp2 bond-bending vibrations involving hydrogen-atom movement in a direction perpendicular to the plane formed by σ bonds of the carbon atom). Other bands originating from OL1000 are observed in the occurrence range of polyurethane-matrix-characteristic bands. 

On the FTIR spectrum of PUR–OP550 material (Figure S2), additional signals with a maximum at 1030 cm^−1^ were observed, related to the stretching vibrations of C–O groups as parts of clusters coming from OP550 phosphoric polyol, as well as additional signals in the out-of-plane area (<1000 cm^−1^) and differences in the shape of the band in the 1330–1170 cm^−1^ range related to the presence of a band with a maximum at 1250 cm^−1^ in OP550. Other bands originating from OP550 are observed in the occurrence range of polyurethane-matrix-characteristic bands. FTIR spectra of the reference foam, foam modified with OP550 and APP flame retardants (PUR–OP550_APP) and OP550 and APP, additives are shown in Figure S3. In the FTIR spectrum of PUR–OP550_APP material, in addition to the changes visible in the PUR–OP550 spectrum, additional signals were observed in the <600 cm^−1^ area, related to the introduction of APP. Other bands coming from flame retardants occur in the occurrence range of the polyurethane-matrix-characteristic bands. Figure S4 summarises the FTIR spectra of the reference foam, foam modified with flame retardants—OP550 and expandable graphite (PUR–OP550_graphite), and OP550 additive. In the FTIR spectrum of PUR–OP550_graphite, identical changes related to the introduction of OP550 polyol were observed in PUR–OP550 foam. No additional signals related to graphite introduction were observed. Other bands from OP550 or signals from graphite are observed in the occurrence range of polyurethane-matrix-characteristic bands. In the FTIR spectrum of PUR–Fyrol PNX LE (Figure S5), additional signals with a maximum at 1025 cm^−1^ were observed, related to the stretching vibrations of C–O groups as components of clusters originating from Fyrol PNX LE, as well as additional signals in the 1330–1170 cm^−1^ range related to the presence of a band with a maximum at 1266 cm^−1^ in Fyrol PNX LE. Other bands originating from Fyrol PNX LE were observed in the occurrence range of polyurethane-matrix-characteristic bands. In the FTIR spectrum of PUR–Fyrol HF5 (Figure S6), additional signals with a maximum at 1485 cm^−1^, 1030 cm^−1^ and in the out-of-plane area (<1000 cm^−1^) were observed, as well as differences in the shape of the bands in the 1330–1170 cm^−1^ range related to the introduction of flame retardant. Other bands originating from Fyrol HF5 were observed in the occurrence range of polyurethane-matrix-characteristic bands.

**Figure 4:**
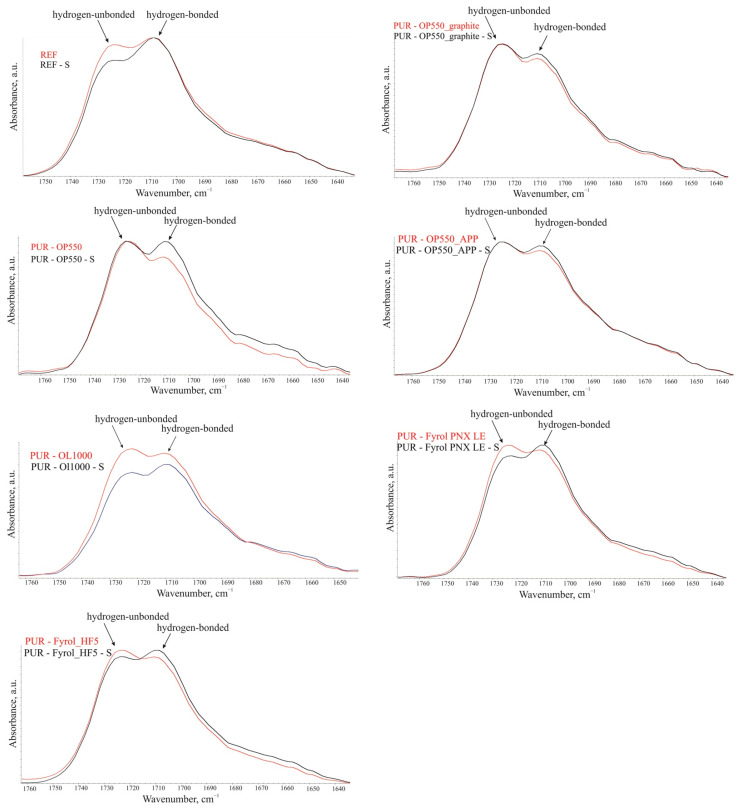
Summary of the 1770–1630 cm^−1^ range spectra fragments.

Differences were observed in the multiplet signal shape in the wavenumber range of 1760–1640 cm^−1^ for FTIR spectra obtained for the samples cut out from the foam core and skin. The differences were related to the change in the intensity of components originating from the stretching vibrations of urethane domains carbonyl groups, both bound and unbound with a hydrogen bond (Figure 4) [37]. The results published by Pretsch et al. [37] indicate that signals related to the carbonyl stretching vibrations of free and hydrogen-bonded groups were located at 1728 cm^−1^ and 1702 cm^−1^, respectively. Comparison of the signal shape in the range of 1770–1640 cm^−1^, characterized by peaks with two maxima, indicates that the ratio of the signal intensity with the maximum at lower wavenumber values to the signal maximum at higher wavenumber values is higher for foams cut from the skin of foams. This indicates a higher share of bound carbonyl groups in the samples cut out from the foam skin, revealing a higher degree of phase separation in the rigid segments in those samples. Differences in described signal shape were observed for all analysed foams.

### 3.6. Differential Scanning Calorimetry

**Figure 5:**
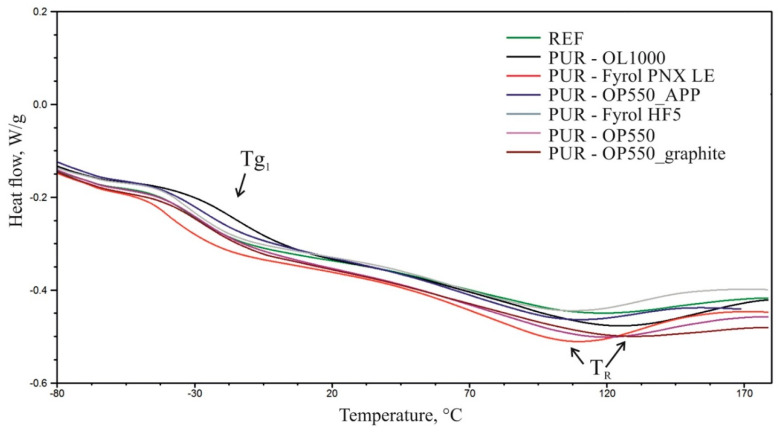
Summary of thermograms obtained during the first heating of the samples.

**Figure 6:**
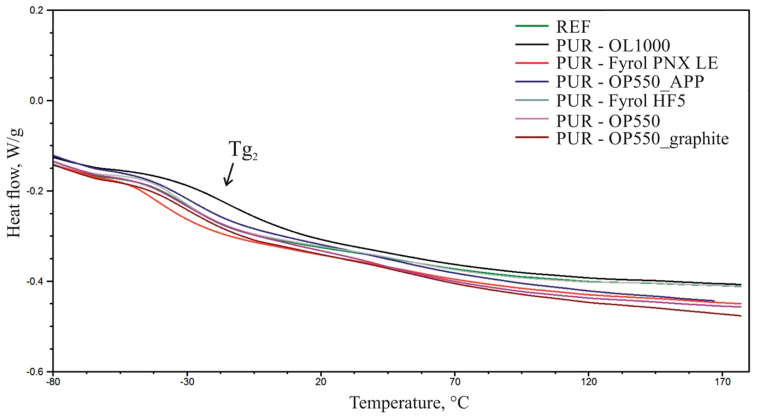
Summary of thermograms obtained during the second heating of the samples.

Based on DSC thermograms, it was observed that during the first heating, an inflexion typical of glass-transition temperature (Tg_1_) occurs on the curves in the soft phase of polyurethane, as well as an endothermal peak in the 120–170 °C temperature range with, an extreme at (T_R_) temperature, resulting from the relaxation occurring in the polyurethane (Figure 5). During the second heating of the sample, only an inflexion typical of the soft phase glass-transition temperature was observed (Tg_2_). Table 6 summarises the identified glass-transition temperatures (Figure 6). Due to the observed relaxation process during the first heating cycle, only the results obtained in the second heating cycle were analysed.

The results reveal that the application of flame retardants affected the soft-phase glass-transition temperature of polyurethane. The introduction of Fyrol PNX LE and Fyrol HF5 contributed to a reduction in the glass-transition temperature of the foams, which suggests a plasticising action of the additives. The addition of OL1000 caused a significant increase in the glass-transition temperature compared to the reference material, which suggests reduced mobility of the soft-phase flexible segments due to introduction of flame retardants. The addition of OP550 and APP at 15 php/15 php caused a minor reduction in the glass-transition temperature. The use of OP550 at 30 php and OP550 and graphite at 15 php/15 php did not affect the glass-transition temperature of the soft phase of the foams.

### 3.7. Thermogravimetric Analysis

**Figure 7:**
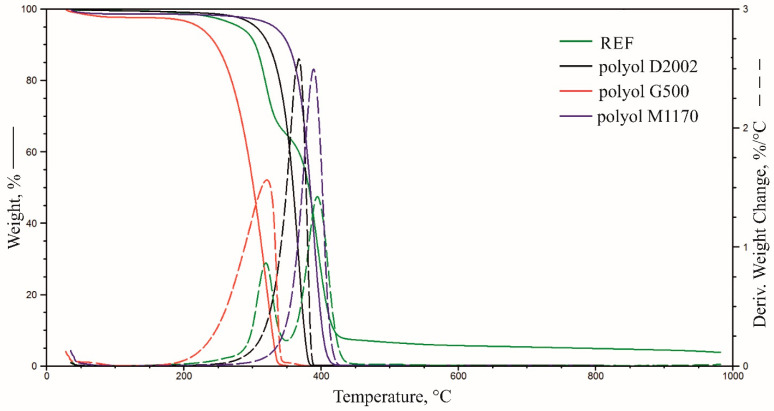
Summary of weight change and derivative weight change curves for the reference material and polyols.

**Figure 8:**
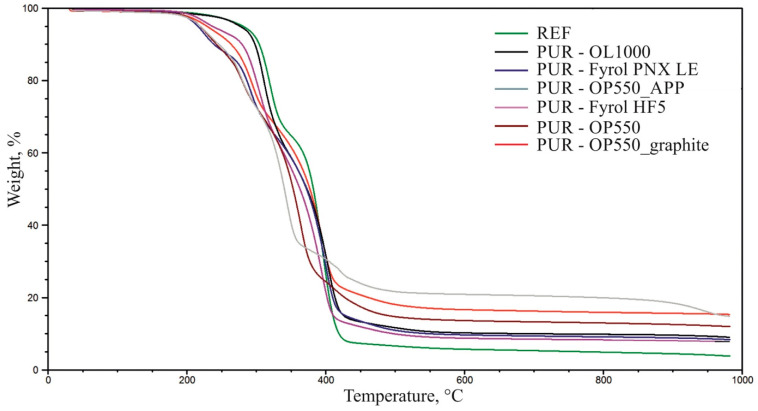
Summary of weight change curves for the foams.

**Figure 9:**
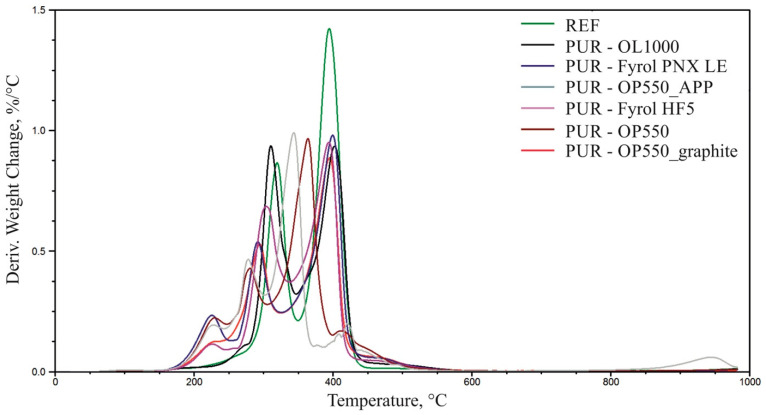
Summary of derivative weight change curves for the foams.

Figure 7, Figure 8 and Figure 9 summarise TG and DTG thermograms for polyols and foams. Table 7 summarises the results of the thermogravimetric analysis.

Thermal degradation of the reference material occurs in two stages, with the maximum degradation rate V_max2_ = 0.86%/°C and V_max3_ = 1.42%/°C at T_max2_ = 319 °C and T_max3_ = 394 °C (Figure 9, Table 6), respectively. The peak, which shows the maximum degradation rate at T_max2,_ is a multiplet band formed due to the thermal degradation of urea and urethane bonds in rigid segments. This stage is also linked to the thermal degradation of elastic segments, confirmed by thermograms describing the thermal stability of the polyols used to make the foams [8,38]. The sample weight loss at this stage amounts to ∆m_2_ = 33%. The band with the maximum degradation rate at T_max3_ = 394 °C was formed due to thermal degradation of elastic segments [8,38]. The sample’s weight loss at this stage amounts to ∆m_2_ = 58%.

The use of flame retardants caused an increase in the number of thermal-degradation stages of the foams and contributed to the gradual degradation of the materials in a broader temperature range than of the reference material. The temperature at which 5% material loss occurred was lowered after introducing flame retardants, which is characteristic for materials with FRs able to decompose at lower temperatures and help create the char. The residue of foams containing flame retardants greater than in the reference material, probably resulting from the formation of a carbon layer during thermal degradation. More detailed charts demonstrating TG and DTG curves of foams and flame retardants are included in the Supplementary Materials (Figures S7–S12).

### 3.8. Compression Set and Rebound Resilience

Durable deformation identified at 50% and 90% compression is an important indicator describing the mechanical resistance of foams during use. The value of durable deformation should not exceed 15% [8]. Analysis results of durable deformations of the foams reveal that all produced materials fulfil the requirements set for viscoelastic foams (Table 8).

The application of flame retardants positively influenced the resistance of foams to mechanical factors, decreasing durable deformation at 50% and 90% compression. The characteristic feature of viscoelastic foams is their low rebound resilience of up to 20% [8]. The analysis results suggest that all tested foams are characterised by elasticity typical of this group of materials. The application of OL1000 significantly reduced the elasticity of the foams, which can result from changes in material structure caused by a significant increase in polyol masterbatch viscosity after FR introduction. The use of Fyrol PNX LE rendered the most elastic foam, and no significant changes were observed in this property after introduction of other flame retardants.

### 3.9. Burning Behaviour

**Figure 10:**
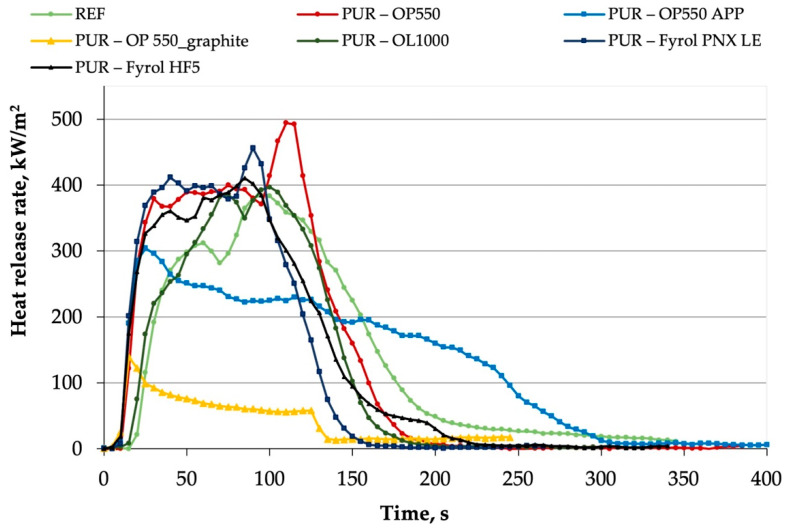
Representative curves of heat release rate obtained for foams modified with flame retardants.

The values of the critical parameters obtained based on the test carried out with a cone calorimeter are summarised in Table 9. Figure 10 shows representative curves of the heat release rates (HRR) in the function of time. Based on the course of the heat-release-rate curves only for systems containing OP550 and APP or expandable graphite, a decrease in the HRR occurred compared to REF foam. HRR curves obtained for PUR–OP550_APP and PUR–OP550_graphite foams after reaching a maximum value at the beginning of the test were lowered and flattened, which suggests the formation of an insulating layer on the material surface, protecting the material against heat flux. 

Time to ignition (TTI) for the reference foam amounted to 21 s, while for the materials containing flame retardants, it was reduced to a dozen seconds. The highest TTI value of all samples modified with flame retardants was observed for the foam with OL1000, which amounted to 18 s. A relatively short time to ignition is caused by a structure of foamed materials and their low thermal conductivity. The cell walls are very thin and reach the ignition temperature quickly because of poor heat conduction into the material [39].

For the maximum value of the peak of heat release rate (pHRR), the critical parameter of fire hazard posed by materials, the lowest values were determined for PUR–OP550 APP and PUR–OP550_graphite foams. A value of 138 kW/m^2^, which is nearly three times lower than that for REF foam, was obtained for the sample modified with OP550 and expandable graphite. A value decrease due to the introduction of flame retardant systems that contain OP550 and ammonium polyphosphate or graphite was also observed for the maximum average rate of heat emission (MARHE). MARHE is a quantitative description of the material influence of flame propagation [39], and its value strongly depends on the HRR curve course. There was either no significant influence or an increase in the pHRR and MARHE compared to the reference foam for other materials. As an explanation of behaviour for PUR foams containing flame retardants, such as Fyrol PNX LE, Günther et al. [39] indicated two-stage combustion of this type of material. Fyrol PNX LE contains significant amounts of phosphorus and operates mainly in the gaseous phase, but its low quantity remains in the condensed phase. The first stage, represented in the HRR plot as a rapid increase and flattening of the curve between 10 and 90 s, covers the material ignition and evaporation of the flame retardant, which inhibits fire. In a liquid condensed phase, formed due to material melting and loss of its primary structure, polyol and small amounts of isocyanate residues are combusted at the next stage. The process is intensified by additional reaction products coming from Fyrol PNX LE, and since the entire mass of the condensed phase burns simultaneously, a rapid increase in HRR is observed. Such a burning behaviour causes foams modified with Fyrol PNX LE to perform favourably in flammability tests (e.g., oxygen index, UL-94) other than the cone calorimeter, where the process is inhibited in the first stage. In turn, when graphite is added, the burning behaviour of the foam changes, and structural collapse is prevented [20]. Graphite works in the solid phase, forming a layer of low-density char on the sample’s surface and dramatically reducing smoke emission. The reaction of sulfuric acid with graphite flakes, which expands its volume by about 100 times, is accompanied by gases generated at high temperatures (CO_2_, H_2_O, SO_2_) [40].

For total heat release (THR), values lower than for the REF sample were obtained for most samples, except for PUR–OP550 and PUR–OP550_APP foams, and the lowest value was again reported for the system with expandable graphite (reduction by 78%). THR reduction suggests incomplete combustion caused by reduced capacity, confirmed by the value of the effective heat of combustion (EHC) or the formation of a carbonaceus layer [39]. All applied flame retardants were observed to reduce EHC, suggesting activity of flame retardants in the gaseous phase. Unfortunately, such a mechanism of inhibition of burning under forced flaming fire conditions, which are quite common with organophosphorus flame retardants, is often accompanied by an increase in smoke emission and release of hazardous carbon dioxide [37].

Smoke containing incomplete combustion products, next to suffocating and irritating gases, significantly increases the number of victims. Smoke emissions from the analysed materials were evaluated with specific extinction area (SEA) and total smoke release (TSR). The SEA parameter increased due to the introduction of flame retardant, while the lowest value, which was 9% higher than for the REF foam, was determined for the PUR OP550_graphite system. The lowest TSR value was also reported for this material, and it was twice as low as the value obtained for the material that did not contain flame retardants. Other agents or systems significantly increase smoke emission, confirming activity of most flame retardants in the gaseous phase.

**Figure 11:**
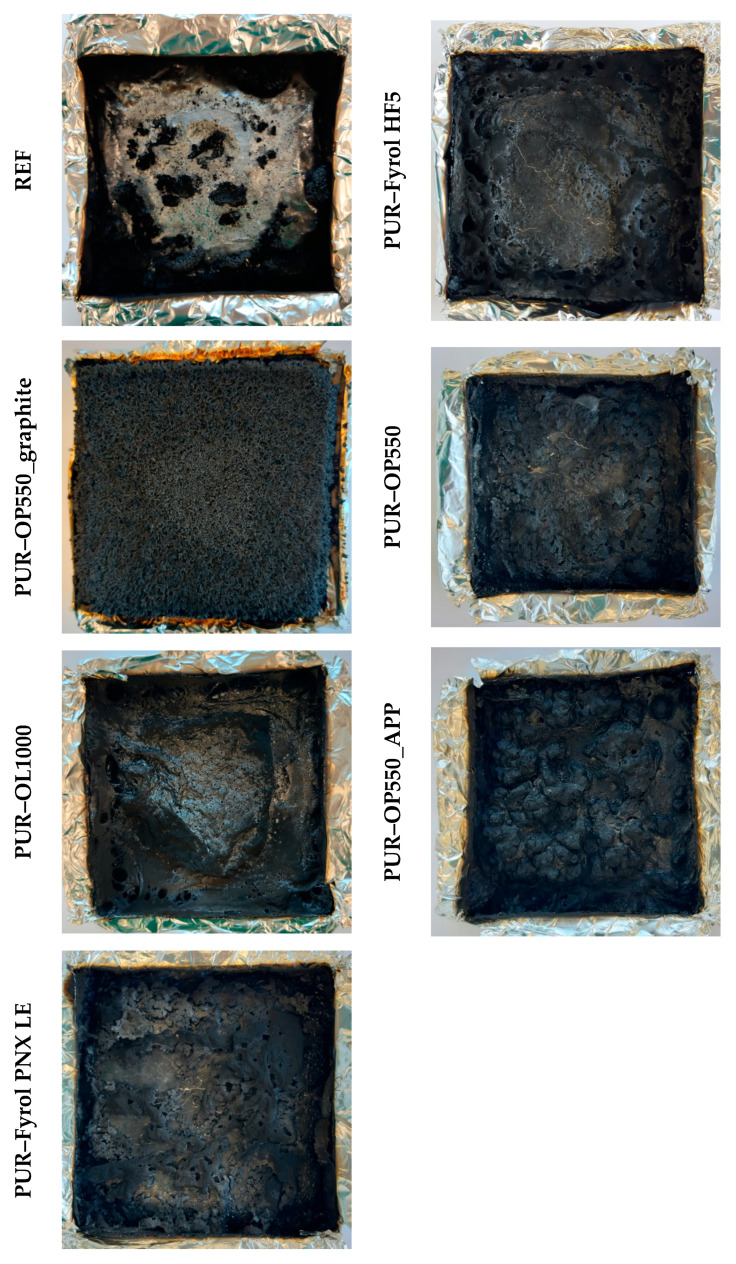
Foam samples after cone-calorimeter tests.

Figure 11 presents photos of samples after cone-calorimeter tests. The REF foam was almost completely burned out, while for the remaining samples, the formation of char was observed, the amount and appearance of which depended on the type of flame retardant. However, the formation of a layer with a thickness ensuring an appropriate temperature gradient was observed only in the case of foam containing expanded graphite. Its appearance is characteristic of the burning residue of materials modified with this flame retardant. The remaining samples formed only discontinuous layers, which could be caused by an insufficient amount of the condensed phase capable of charring and the release of gaseous products.

**Figure 12:**
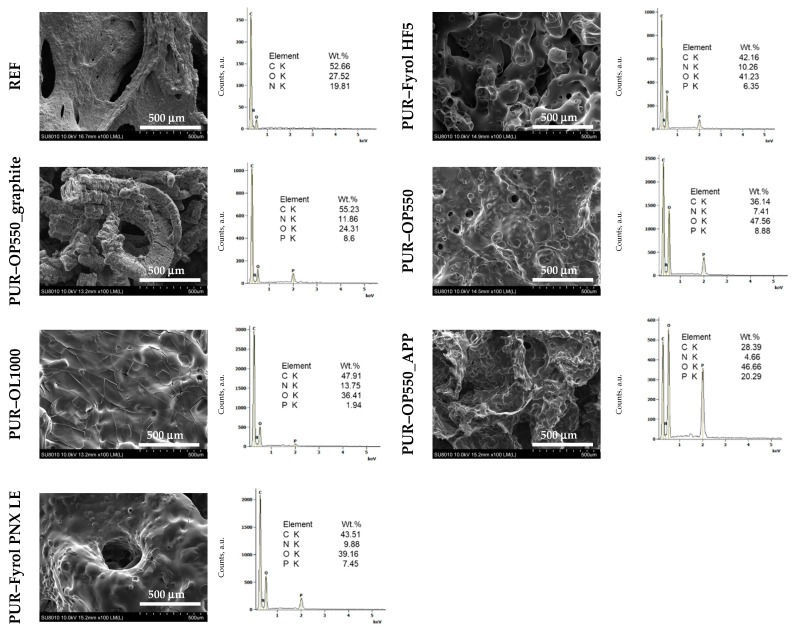
Scanning electron microscope images and energy dispersive spectroscopy results for foam samples after cone calorymetry tests.

The morphology of the residues was investigated by SEM (Figure 12). Since the residues of most samples, excluding foam modified with graphite, was relatively thin and/or exhibited holes, they provided only partial protection. Chemical composition obtained by SEM/EDS indicates that residues were comprised of C, O, and N, as well as P elements, in the case of foams modified with flame retardants. This suggests that FRs could decompose into the products that participate in the crosslinking process, affecting a more compact char [40].

### 3.10. Cytotoxicity of Polyurethane Foam Extracts

**Figure 13:**
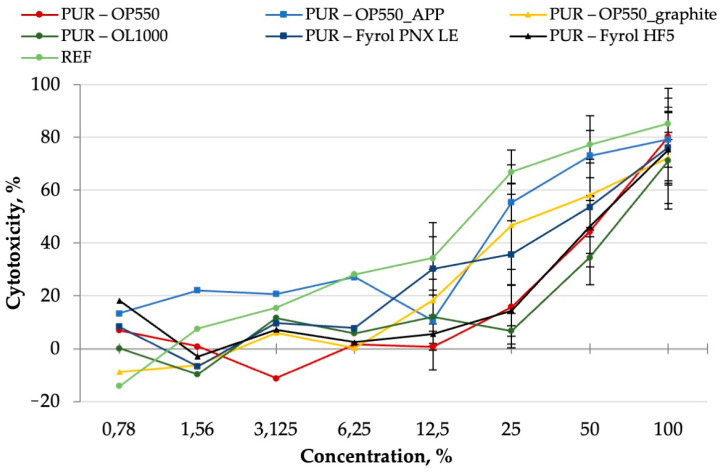
Cytotoxicity of PUR extracts after the 24-h exposure of human keratinocyte cell line HaCaT in neutral red uptake assay. Each point represents the mean absorbance values of the four replicates (±SD) from three independent experiments.

Human keratinocyte cells HaCaT were exposed to water-soluble polyurethane foam fractions at concentrations ranging from 0.78 to 100% for 24 h. The curves showing the cytotoxicity of the tested foams are presented in Figure 13 as the mean ± SD of three independent experiments. A statistical analysis of the obtained results is presented in Table S1. The cytotoxicity of the foam extracts depended on their type and was directly proportional to the concentration. Generally, all samples showed an increase in cytotoxicity from a concentration of 12.5%. It was observed that the high concentrations of foam extracts resulted in high cytotoxicity. The highest cytotoxicity characterised the sample marked as REF (*p* < 0.05), while the sample marked as PUR–OL1000 was the least cytotoxic (*p* < 0.05).

These results are in agreement with the IC_50_ values. Table 10 shows the IC_50_ values for each sample. The foam extract marked as REF showed the highest cytotoxicity, for which the IC_50_ value was 18.54%, while for the sample marked as PUR–OL1000, the IC_50_ amounted to 72.12% (the least cytotoxic). The positive control was the most cytotoxic towards HaCaT cells (IC_50_ 2.02%). Non-toxic concentrations (IC_0_) were as follows: OL1000 ≤ 25%; PUR–Fyrol HF5 and PUR–OP550 ≤ 12.5%; PUR–OP550_graphite and PUR–Fyrol PNX LE ≤ 6.25%; REF ≤ 1.56 and PUR–OP550_APP ≤ 0.78%.

**Figure 14:**
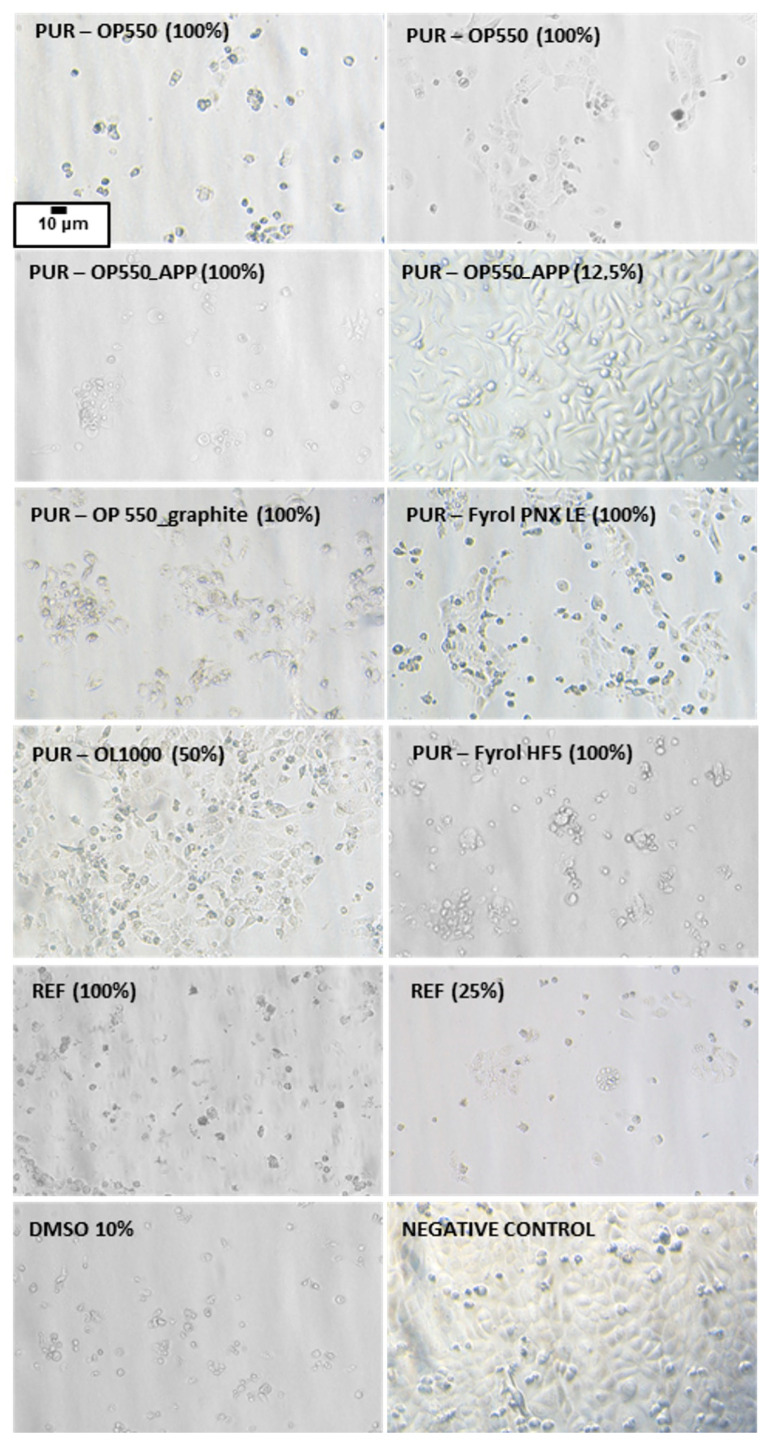
Example microphotographs of human keratinocyte cell line HaCaT after 24-h exposure to PUR extracts. Total magnification: 200× (Nikon Ts2, contrast EMBOSS, Tokyo, Japan).

Morphological changes in the HaCaT cell monolayer in the presence of PUR extracts are presented in Figure 14, while qualitative morphological grading of cytotoxicity according to ISO 10993-5 standard is shown in Table 11. 

The cells in the untreated control (vehicle) formed a regular and homogeneous monolayer with clearly visible cell membranes, cytoplasm, and nuclei; no cell lysis or cell-growth reduction was observed. After exposure to PUR extracts, the monolayer was no longer confluent, the number of cells per visual field decreased compared to the vehicle, and the cells remained detached/semi-detached/loosely attached from/to the substrate surface. In the presence of a 100% concentration of tested PUR foams, severe cytotoxicity and nearly complete destruction of the cell monolayer were observed. The following changes in the morphology of the cells were observed: irregular/round shape, cells shrinkage and lysis, cell membrane fragments and cell residues, chromatin condensation, intracytoplasmic granules, cell swelling, cytoplasmic blebs, strong vacuolisation of cytoplasm, dead cells. Generally, microscopic analysis was consistent with the cytotoxicity results. For qualitative morphological grading of cytotoxicity, it was estimated (for 25% extract concentration) that the positive control and REF induced severe reactivity; PUR–OP550_graphite and PUR–OP550_APP—moderate reactivity; PUR–Fyrol PNX LE—mild reactivity; PUR–Fyrol HF5 and PUR–OP550—slight reactivity; PUR–OL1000—no reactivity (Table 10).

It is essential to search for safe, non-cytotoxic polyurethane materials for biomedical applications. According to the International Agency for Research on Cancer (IARC), PUR foams are assigned to Group 3 as “not classifiable as to its carcinogenicity to humans” [41]. The biological activity and properties of PURs may depend on their chemical composition, synthesis method, technology, preparation, and structure [42]. In our study, REF is unmodified foam, i.e., without flame retardants, which induced the highest cytotoxicity towards HaCaT cells out of all tested foam extracts. The cytotoxicity of PUR foams can be caused by the leaching of some chemical substances from the foam during extraction, such as dibutyltin dilaurate catalyst [43], which can be neurotoxic and cause DNA damage and injury to animal and mammalian cells in vitro (Swiss 3T3 mouse fibroblasts, human endothelial cells—HEC and 3T3-L1 preadipocytes from embryonic tissue of Swiss mice) [44,45,46]. Król et al. [47] estimated cytotoxic activity of polyurethanes based on two non-aromatic diisocyanates—1,6-hexamethylene diisocyanate (HDI) and isophorone diisocyanate (IPDI)—as well as two kinds of polyester polyols, which are often used as biomedical materials. In direct-contact toxicity assay conducted on normal human fibroblasts (BJ) and HaCaT cells, the authors detected that both cell lines were similarly sensitive to tested PUR, except for one sample, to which higher sensitivity was observed for HaCaT cells. The tested samples demonstrated moderate and even severe cytotoxicity. The diverse cytotoxicity of different PUR samples is probably due to of different chemical and physicomechanical properties. In our study, the addition of flame retardants to foams appears to have reduced cytotoxic activity towards HaCaT cells. PUR–OP550 includes a flame retardant (non-halogenated phosphorus polyol) designed explicitly for PUR foams, which is non-mutagenic, and for which no data on cytotoxicity is available. PUR–Fyrol PNX LE and PUR–Fyrol HF5 foams include proprietary phosphorus-ester-blend retardants, which can be cytotoxic for many human epithelial cell lines, such as hepatocyte carcinoma HepG2, colon adenocarcinoma Caco-2, and alveolar adenocarcinoma A-549 [22]. The authors concluded that these compounds induce cytotoxicity in various cell lines at relatively high concentrations. They suppress cell viability, induce overproduction of reactive oxygen species and DNA damage, and increase leakage of cell enzymes. Different cell lines have different sensitivity and respond differently to phosphorus-ester exposure. Additionally, exceptionally high cytotoxicity was observed in our studies at the highest tested concentrations of PUR foams: 25, 50, and 100%. This is essential because HaCaT keratinocytes are normal cells, which are usually more sensitive to the effects of toxic chemical compounds than cancerous cells. Our cytotoxicity tests demonstrated that the following foams are the safest materials for biomedical applications: PUR–OL1000 as the most appropriate, followed by PUR–Fyrol HF5 and PUR–OP550, with zero or slight cytotoxicity, respectively.

## 4. Conclusions

The development of viscoelastic polyurethane foams intended for application as self-adapting seals of respiratory protective devices was described. Due to the mechanical characteristics of such foams, the adaptation of the shape of seals to the user’s anthropometric dimensions will be possible to eliminate any potential leakage when the user is unaware of the danger or is not able to adjust the RPD’s fit because of the nature of the tasks performed. To ensure low flammability, low cytotoxicity, and good surface wettability, specific changes in polyurethane formulation were made. The physical and chemical characteristics of developed materials were comprehensively investigated.

The results of viscosity analysis reveal that the introduction of flame retardants into a polyol mixture caused an increase in the viscosity of the systems. Only the addition of OL100 resulted in an increase in viscosity, which could adversely affect the technological process at the stage of seal manufacturing. The structure of the foams was uniform, with the mean equivalent diameter ranging from 182 and 255 μm and an oval shape. Total porosity was high and ranged from 74 to 87%. The homogeneity of the foam microstructure translates into the homogeneity of the properties of the final product, which is important due to the target application of foams. The results of chemical-structure analysis revealed a higher content of bound carbonyl groups in the samples cut out from the foam skin than in the samples cut out from the foam core, which indicates a higher degree of phase separation in the rigid segments of the foam skin. 

The application of flame retardants affected the soft-phase glass-transition temperature of polyurethane and caused an increase in the number of thermal degradation stages of the foams. It also contributed to material degradation in a broader temperature range compared to the reference material. Based on tests carried out on a cone calorimeter, it was discovered that a drop in the heat-release-rate value occurred only in two of the analysed flame retardants (PUR–OP550_APP and PUR–OP550_graphite), compared to the reference foam. 

Analysis of the contact angle revealed hydrophilic properties of most foams at the human body temperature. The highest contact-angle values were measured for the reference foam (REF), Fyrol HF5, and graphite-modified samples (PUR–Fyrol HF5, PUR–OP550_graphite). This is a satisfactory result from the point of view of the comfort of using RPDs with self-sealing elements. At low concentrations, all of the foams induced minor or mild cytotoxic action on HaCaT cells after 24 h of exposure. Surprisingly, the application of flame retardants reduced the cytotoxicity in nearly all tested samples, compared to the reference variant (non-modified foam), which offers a prospective use of the developed materials in RPD design. Because technological works aimed to ensure non-flammability, good wettability, and low cytotoxicity of foams used as facepiece seals in respiratory protective devices, further work should focus on formulation optimisation based on OP550 and graphite.

## Figures and Tables

**Table 1:** Characteristics of the flame-retardants.

Designation	Trade Name	Supplier	Description
Graphite	Graphite EG290	Sinograf, Toruń, Poland	Flake graphite with a carbon content of approx. 90%, expansion degree 200–300 mL/g, and bulk density of 0.66 g/cm^3^, and average grain size of 352 μm
OP550	Exolit OP550	Clariant, Łódź, Poland	Reactive halogen-free phosphor flame retardant with functionality 2
OL1000	Nofia OL1000	WTH Walter Thieme Handel GmbH, Stade, Germany	Halogen-free flame retardant with 10.4 wt% phosphor content
Fyrol PNX LE	Fyrol^®^ PNX LE	ICL Industrial Products, Bitterfeld-Wolfen, Germany	Halogen-free phosphor flame retardant
Fyrol HF5	Fyrol^®^ HF5	ICL Industrial Products, Bitterfeld-Wolfen, Germany	Halogen-free phosphorus ester flame retardant
APP	Addforce FR APP201	WTH Walter Thieme Handel GmbH, Stade, Germany	Ammonium polyphosphate with 31.39 wt% and 14.69 wt% of phosphor and nitrogen content, respectively

**Table 2:** Weight ratios of flame-retardants in foam formulations and mixtures for viscosity testing.

Foam/Mixture Type	Flame Retardant Content, php					
	Exolit OP550	Graphite	OL1000	Fyrol PNX LE	Fyrol HF5	APP
REF	0	0	0	0	0	0
PUR–OP550_graphite	15	15	0	0	0	0
PUR–OL1000	0	0	30	0	0	0
PUR–Fyrol PNX LE	0	0	0	20	0	0
PUR–Fyrol HF5	0	0	0	0	20	0
PUR–OP550	30	0	0	0	0	0
PUR–OP550_APP	15	0	0	0	0	15

**Table 3:** Apparent density of foam samples by flame-retardant type.

Foam Type	Apparent Density, g/cm^3^
REF	120
PUR–OP550_graphite	165
PUR–OL1000	156
PUR–Fyrol PNX LE	153
PUR–Fyrol HF5	149
PUR–OP550	170
PUR–OP550_APP	181

**Table 4:** Structural parameters of foams by flame-retardant type.

Foam Type	Mean Pore Equivalent Diameter *, d_2_, μm	Pore Aspect Ratio AR, a.u.	Total Porosity, %
REF	211 ± 119	1.32 ± 0.18	82 ± 1.03
PUR–OP550_graphite	209 ± 113	1.29 ± 0.18	78 ± 1.11
PUR–OL1000	182 ± 153	1.30 ± 0.18	76 ± 1.69
PUR–Fyrol PNX LE	225 ± 147	1.27 ± 0.14	76 ± 1.25
PUR–Fyrol HF5	208 ± 131	1.30 ± 0.16	74 ± 1.87
PUR–OP550	249 ± 115	1.27 ± 0.16	82 ± 1.16
PUR–OP550_APP	255 ± 127	1.28 ± 0.16	87 ± 1.01

* mean ± standard deviation.

**Table 5:** Contact angle and surface energy.

Foam Type	Contact Angle, °						Surface Energy, mJ/m^2^		
	Distilled Water		Acidic Sweat		Alkaline Sweat				
	NC	37 °C	NC	37 °C	NC	37 °C	$\gamma_{s}^{p}$	$\gamma_{s}^{d}$	$\gamma_{s}$
REF	85.8 ± 1.8	95.2 ± 0.7	96.3 ± 4.5	97.5 ± 3.2	85.8 ± 4.6	96.2 ± 6.6	41.75	22.34	64.09
PUR–OP550_graphite	81.1 ± 0.6	89.1 ± 2.1	90.3 ± 0.6	95.3 ± 2.1	87.9 ± 0.6	90.1 ± 2.6	39.34	23.57	62.91
PUR–OL1000	73.4 ± 1.3	83.8 ± 1.6	84.9 ± 1.6	88.0 ± 4.3	75.9 ± 1.5	84.8 ± 3.6	29.96	6.13	36.09
PUR–Fyrol PNX LE	77.3 ± 1.6	87.0 ± 1.9	88.2 ± 2.6	87.5 ± 1.4	94.2 ± 2.6	88.0 ± 4.6	35.31	8.23	43.54
PUR–Fyrol HF5	87.0 ± 1.9	94.2 ± 3.3	95.4 ± 3.6	96.4 ± 1.6	95.2 ± 3.6	95.2 ± 5.6	45.89	26.2	72.09
PUR–OP550	70.5 ± 3.9	77.0 ± 1.4	78.2 ± 1.6	81.0 ± 2.2	73.7 ± 1.6	78.1 ± 0.6	28.9	11.8	40.7
PUR–OP550_APP	55.7 ± 1.1	64.1 ± 1.4	65.2 ± 1.1	68.0 ± 0.5	66.4 ± 1.1	65.0 ± 1.6	21.68	32.18	53.86

NC—normal conditions.

**Table 6:** Glass-transition temperatures of the foams.

Foam Type	Tg_1_, °C	Tg_2_, °C
REF	−32 ± 1	−32 ± 2
PUR–OP550_graphite	−32 ± 4	−32 ± 1
PUR–OL1000	−14 ± 2	−16 ± 1
PUR–Fyrol PNX LE	−39 ± 2	−41 ± 1
PUR–Fyrol HF5	−34 ± 3	−34 ± 2
PUR–OP550	−29 ± 1	−32 ± 0
PUR–OP550_APP	−27 ± 2	−29 ± 0

**Table 7:** Foam parameters determined from thermogravimetric (TG) and derivative thermogravimetric (DTG) curves.

Foam Type	T_5%_,°C	T_max1_, °C (V_max1_, %/°C)	∆m_1_, % (Range, °C)	T_max2_, °C (V_max2_, %/°C)	∆m_2_, % (Range, °C)	T_max3_, °C (V_max3_, %/°C)	∆m_3_, % (Range, °C)	T_max4_,°C (V_max4_, %/°C)	∆m_4_, % (Range, °C)	T_max5_, °C (V_max5_, %/°C)	∆m_5_, % (Range, °C)	P_600_/P_950_, %
REF	281	-	-	319 (0.86)	32.64 (240–350)	394 (1.42)	57.5 (350–440)	-	-	-	-	5.72/ 3.80
PUR–OP550_graphite	229	228 (0.125)	5.4 (150–240)	294 (0.53)	24.72 (240–325)	395 (0.89)	47.5 (325–440)	466 (0.059)	4.59 (440–600)	-	-	16.66/ 15.34
PUR–OL1000	276	-	4.2 (150–274)	310 (0.94)	34.55 (274–345)	402 (0.93)	47.1 (345–447)	-	3.12 (447–600)	-	-	10.23/ 9.02
PUR–Fyrol PNX LE	216	225 (0.234)	11.8 (140–252)	292 (0.54)	22.77 (252–331)	399 (0.98)	51.1 (330–441)	467 (0.06)	4.11 (440–600)	-	-	9.67/ 8.37
PUR–Fyrol HF5	237	226 (0.115)	5.6 (150–240)	304 (0.69)	32.29 (255–335)	394 (0.95)	48.4 (255–430)	462 (0.05)	3.88 (440–600)	-	-	8.76/ 7.52
PUR–OP550	219	229 (0.224)	9.7 (150–244)	280 (0.43)	15.51 (244–305)	364 (0.96)	46.3 (305–398)	411 (0.17)	11.01 (398–600)	-	-	13.65/ 11.98
PUR–OP550_ APP	219	227 (0.194)	8.6 (150–240)	278 (0.46)	18.46 (240–300)	343 (0.99)	38.5 (300–370)	420 (0.20)	12.10 (370–550)	943 (0.06)	5.1 (800–1000)	20.89/ 14.83

**Table 8:** Rebound resilience and compression set at 50% and 90%, depending on the flame retardant.

Foam Type	Compression Set at 50% (22 h, 70 °C), %	Compression Set at 90% (22 h, 70 °C), %	Rebound Resilience, %
REF	8 ± 2	15 ± 2	11
PUR–OP550_graphite	4 ± 1	7 ± 1	8
PUR–OL1000	2 ± 1	5 ± 3	2
PUR–Fyrol PNX LE	3 ± 2	6 ± 1	18
PUR–Fyrol HF5	2 ± 2	10 ± 2	12
PUR–OP550	5 ± 2	7 ± 3	8
PUR–OP550 APP	1 ± 2	4 ± 2	15

**Table 9:** Foams burning parameters depending on the flame retardant.

Designation	TTI, s	pHRR, kW/m^2^	MARHE, kW/m^2^	THR, MJ/m^2^	EHC, MJ/kg	SEA, m^2^/kg	TSR, m^2^/m^2^
REF	21 ± 3	400 ± 30	262 ± 5	46 ± 2	22 ± 1	255 ± 18	551 ± 16
PUR–OP550_graphite	11 ± 1	138 ± 4	80 ± 3	10 ± 1	13 ± 1	279 ± 40	234 ± 16
PUR–OL1000	18 ± 2	407 ± 19	266 ± 6	41 ± 1	17 ± 0	686 ± 15	1690 ± 25
PUR–Fyrol PNX LE	13 ± 1	452 ± 7	337 ± 2	45 ± 2	19 ± 0	590 ± 9	1413 ± 61
PUR–Fyrol HF5	12 ± 1	410 ± 4	311 ± 3	24 ± 2	18 ± 0	619 ± 2	1533 ± 83
PUR–OP550	13 ± 1	485 ± 31	340 ± 8	52 ± 0	18 ± 0	546 ± 4	1582 ± 9
PUR–OP550 APP	13 ± 1	306 ± 4	219 ± 1	51 ± 1	17 ± 0	632 ± 38	1221 ± 126

TTI—time to ignition; pHRR—peak of heat release rate; MARHE—maximum average rate of heat emission; THR—total heat release; EHC—effective heat of combustion; SEA—specific extinction area; TSR—total smoke release.

**Table 10:** IC_50_ values of foam extracts after the 24-h exposure of human keratinocyte cell line HaCaT evaluated based on cytotoxicity curves.

Sample	IC_50_, %	Cytotoxicity
Positive control (DMSO)	2.02	the highest
REF	18.54	1—the highest
PUR–OP550_graphite	32.11	3
PUR–OL1000	72.12	7—the lowest
PUR–Fyrol PNX LE	45.06	4
PUR–Fyrol HF5	56.69	5
PUR–OP550	58.00	6
PUR–OP550_APP	23.52	2

**Table 11:** Qualitative morphological grading of cytotoxicity of PUR foam extracts (25% concentrations) according to ISO 10993-5 observed in an inverted microscope before adding neutral red.

Sample	Grade	Reactivity	Conditions of All Cultures According to ISO 10993-5
Vehicle control	0	none	Discrete intracytoplasmic granules, no cell lysis, no reduction in cell growth
Positive control (DMSO)	4	severe	Nearly complete or complete destruction of the cell layer
REF	4	severe	Nearly complete or complete destruction of the cell layer
PUR–OP550_graphite	3	moderate	No more than 70% of the cell layers contain rounded cells or are lysed; cell layers not completely destroyed, but more than 50% growth inhibition observed
PUR–OL1000	0	none	Discrete intracytoplasmic granules, no cell lysis, no reduction in cell growth
PUR–Fyrol PNX LE	2	mild	No more than 50% of the cells are round, devoid of intracytoplasmic granules; no extensive cell lysis; not more than 50% growth inhibition observable
PUR–Fyrol HF5	1	slight	No more than 20% of the cells are round, loosely attached, and without intracytoplasmic granules or show changes in morphology; occasional lysed cells are present; only slight growth inhibition observable
PUR–OP550	1	slight	No more than 20% of the cells are round, loosely attached, and without intracytoplasmic granules or show changes in morphology; occasional lysed cells are present; only slight growth inhibition observable
PUR–OP550_APP	3	moderate	No more than 70% of the cell layers contain rounded cells or are lysed; cell layers not completely destroyed, but more than 50% growth inhibition observed

## Data Availability

The data presented in this study are available on request from the corresponding author.

## Supplementary Materials

The following are available online at https://www.mdpi.com/article/10.3390/ma15010151/s1, Figure S1: Fourier transform infrared spectroscopy spectra of REF and PUR–OL1000 foams and the OL1000 flame retardant, Figure S2: Fourier transform infrared spectroscopy spectra of REF and PUR–OP550 foams and the OP550 flame retardant, Figure S3: Fourier transform infrared spectroscopy spectra of REF and PUR–OP550_APP foams and the OP550 and APP flame retardants, Figure S4: The FTIR spectra of REF and PUR–OP550_graphite foams and the expandable graphite and OP550 flame retardant, Figure S5: Fourier transform infrared spectroscopy spectra of REF and PUR–Fyrol PNX LE foams and the Fyrol PNX LE flame retardant, Figure S6: Fourier transform infrared spectroscopy spectra of REF and PUR–Fyrol HF5 foams and the Fyrol HF5 flame retardant, Figure S7: Weight change curves and derivative weight change curves of REF and PUR–OP550_graphite foams and expandable graphite and OP550 flame retardant, Figure S8: Weight change curves and derivative weight change curves of REF and PUR–OL1000 foams and OL1000 flame retardant, Figure S9: Weight change curves and derivative weight change curves of REF and PUR–Fyrol PNX LE foams and Fyrol PNX LE flame retardant, Figure S10: Weight change curves and derivative weight change curves of REF and PUR–Fyrol HF5 foams and Fyrol HF5 flame retardant, Figure S11: Weight change curves and derivative weight change curves of REF and PUR–OP550 foams and OP550 flame retardant, Figure S12: Weight change curves and derivative weight change curves of REF and PUR–OP550_APP foams and OP550 and APP flame retardants, Table S1: Cytotoxicity of polyurethane foams water extracts towards normal immobilized human keratinocyte cells HaCaT after 24 h exposure as measured in Neutral Red Uptake (NRU) assay. Each value represents the mean of four individual replications from three independent experiments (± SD). Superscript letters mean results significantly different (ANOVA, *p* < 0.05) when comparing the same concentrations.
