# Supplementary material for: Viscoelastic Polyurethane Foams with Reduced Flammability and Cytotoxicity
Source: Materials (Basel). 2021 Dec 26;15(1):151. doi: 10.3390/ma15010151 (PMC8746040; doi:10.3390/ma15010151)
Supplement: Supplementary file 1 [file materials-15-00151-s001.zip › materials-1454776-SI.pdf]

materials MDPI

Supplementary Material

Viscoelastic Polyurethane Foams with Reduced Flammability and Cytotoxicity

Małgorzata Okrasa [1,*], Milena Leszczyńska [2], Kamila Sałasińska [2,3], Leonard Szczepkowski [4], Paweł Kozikowski [3], Adriana Nowak [5], Justyna Szulc [5], Agnieszka Adamus-Włodarczyk [1], Michał Gloc [2], Katarzyna Majchrzycka [1] and Joanna Ryszkowska [2]

1 Department of Personal Protective Equipment, Central Institute for Labour Protection—National Research Institute, Wierzbowa 48, 90-133 Łódź, Poland; agada@ciop.lodz.pl (A.A.-W.); kamaj@ciop.lodz.pl (K.M.)
2 Faculty of Materials Science and Engineering, Warsaw University of Technology, Wołoska 141, 02-507 Warszawa, Poland; milena.leszczynska.dokt@pw.edu.pl (M.L.); kamila.salasinska@pw.edu.pl (K.S.); michal.gloc.wim@pw.edu.pl (M.G.); joanna.ryszkowska@pw.edu.pl (J.R.)
3 Department of Chemical, Aerosol and Biological Hazards, Central Institute for Labour Protection—National Research Institute, Czerniakowska 16, 00-701 Warszawa, Poland; pakoz@ciop.pl
4 FAMPUR Adam Przekurat Company, Gersona 40/30, 83-305 Bydgoszcz, Poland; leonardosz@interia.pl
5 Department of Environmental Biotechnology, Lodz University of Technology, 90-530 Łódź, Poland; adriana.nowak@p.lodz.pl (A.N.); justyna.szulc@p.lodz.pl (J.S.)
* Correspondence: maokr@ciop.lodz.pl; Tel.: +48-(42)-6480223

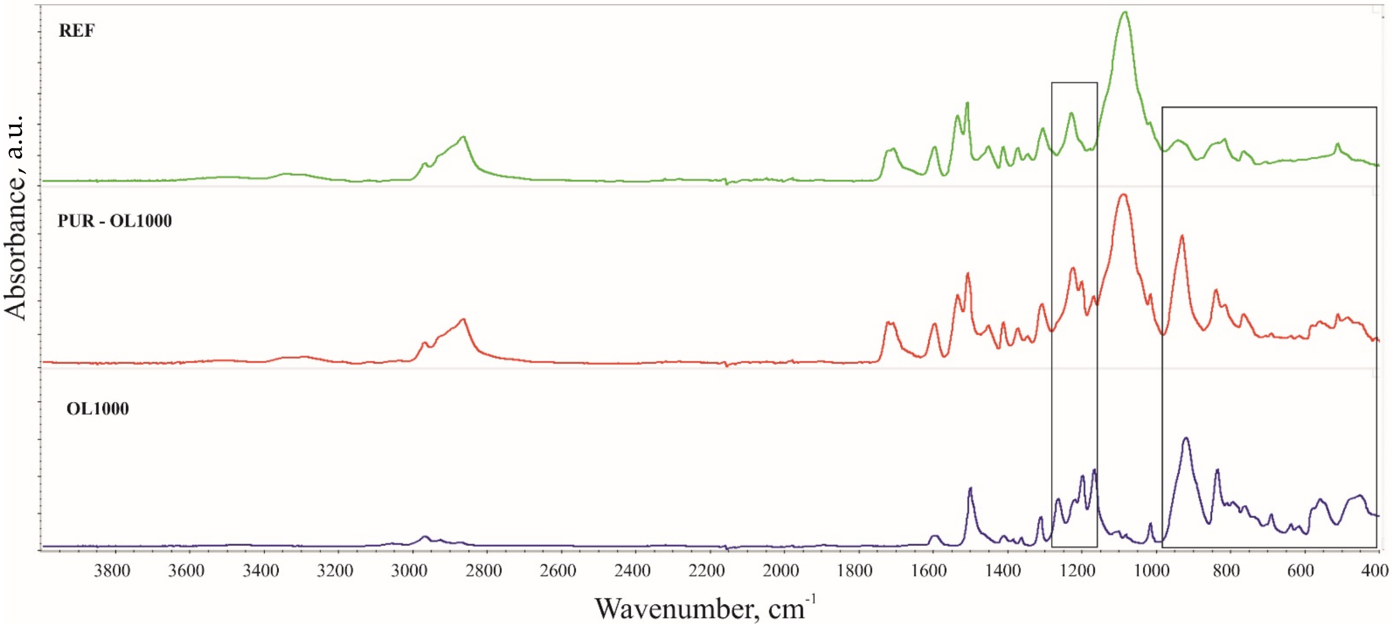

Figure S1. Fourier transform infrared spectroscopy spectra of REF and PUR- OL1000 foams and the OL1000 flame retardant.

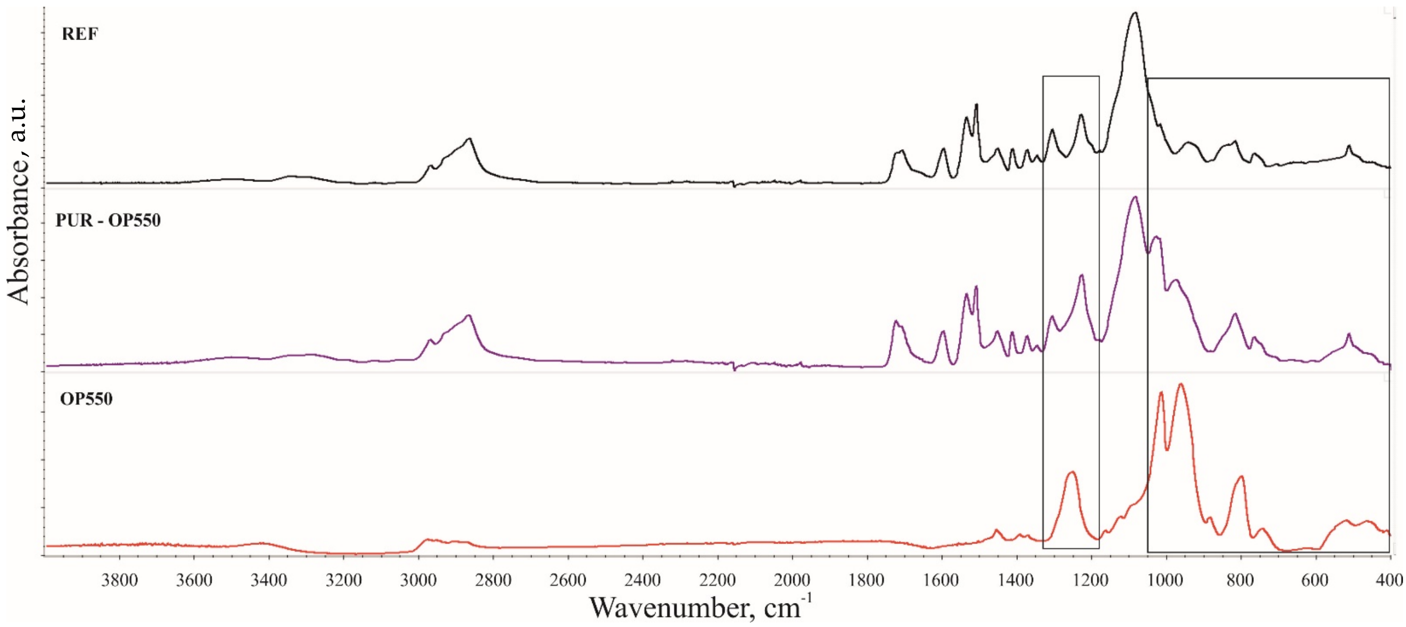

Figure S2. Fourier transform infrared spectroscopy spectra of REF and PUR- OP550 foams and the OP550 flame retardant.

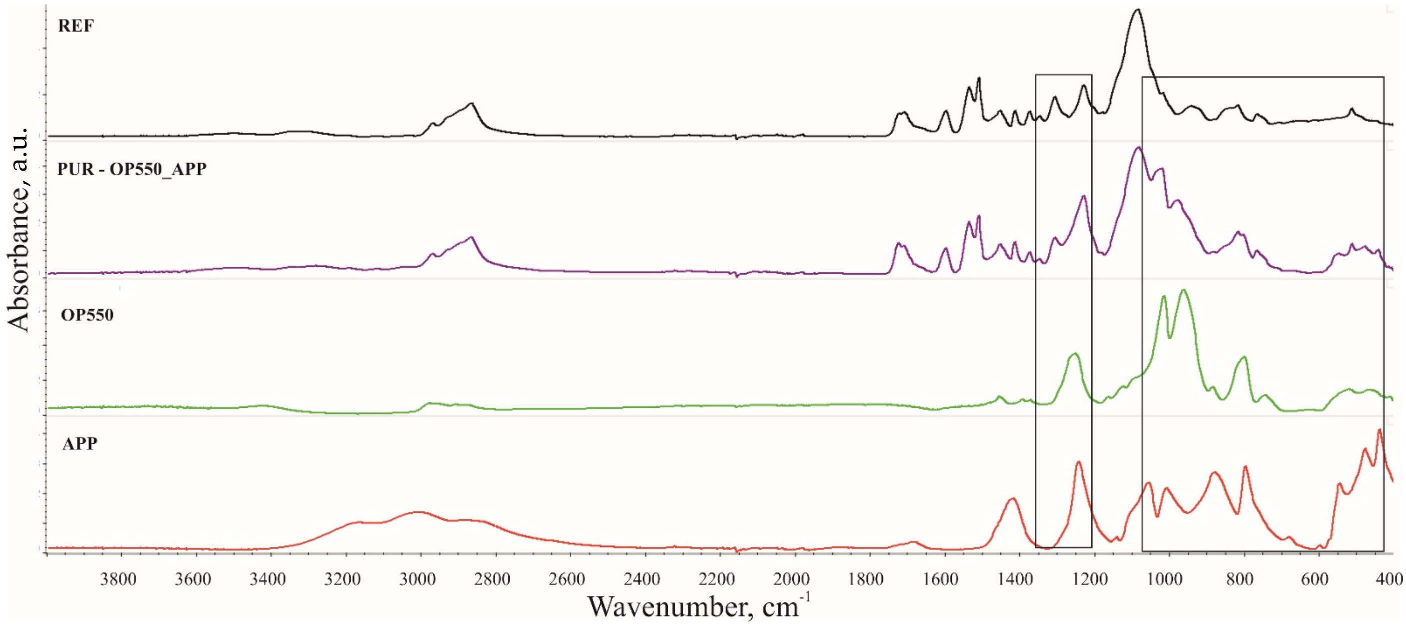

Figure S3. Fourier transform infrared spectroscopy spectra of REF and PUR- OP550_APP foams and the OP550 and APP flame retardants.

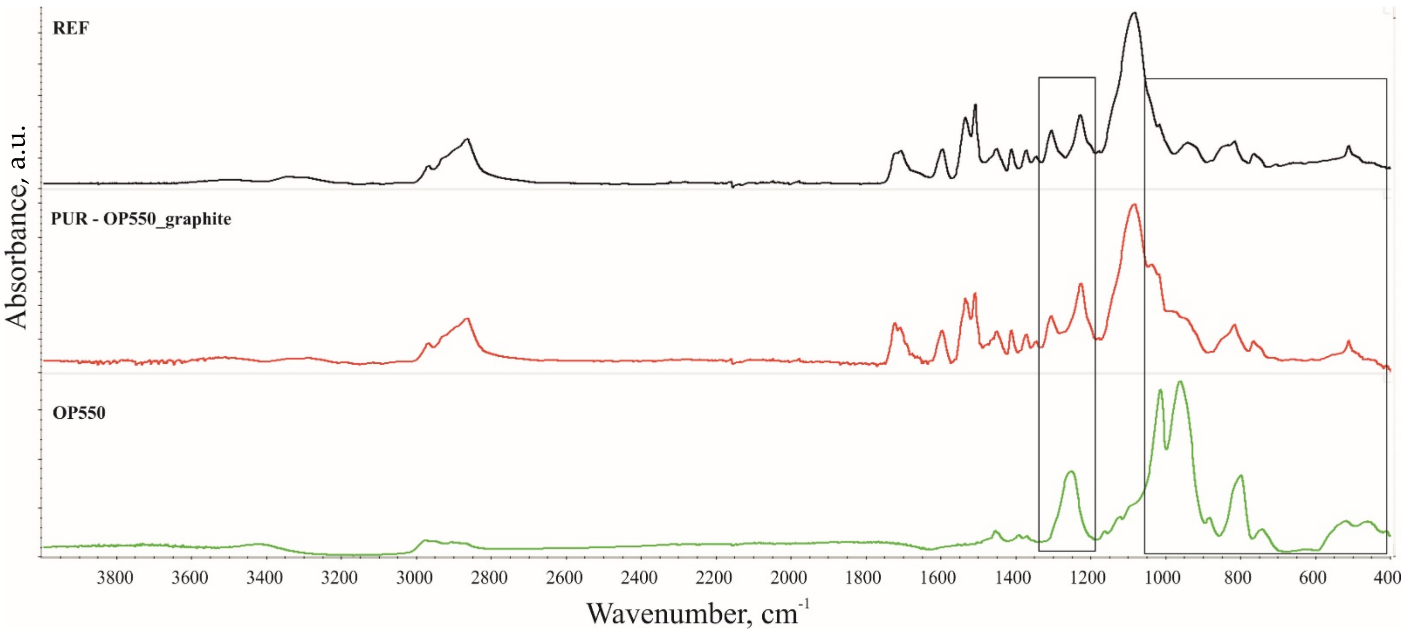

Figure S4. Fourier transform infrared spectroscopy spectra of REF and PUR- OP550_graphite foams and the expandable graphite and OP550 flame retardant.

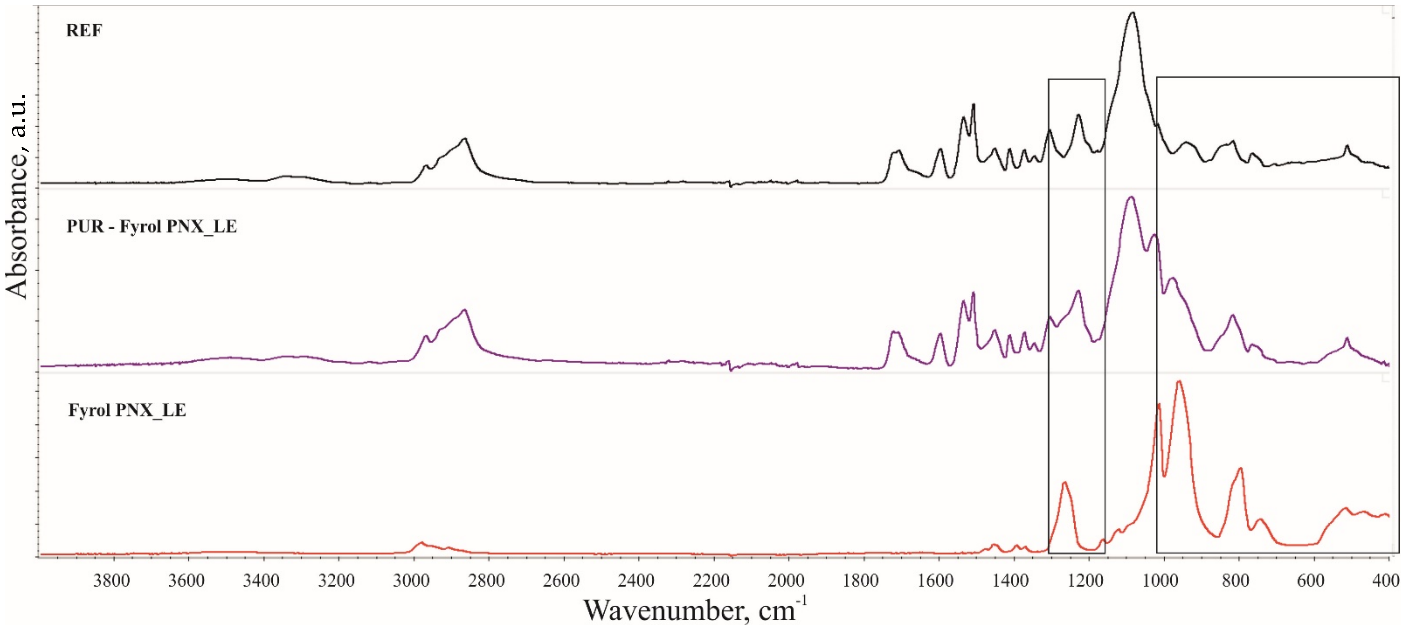

Figure S5. Fourier transform infrared spectroscopy spectra of REF and PUR- Fyrol PNX LE foams and the Fyrol PNX LE flame retardant.

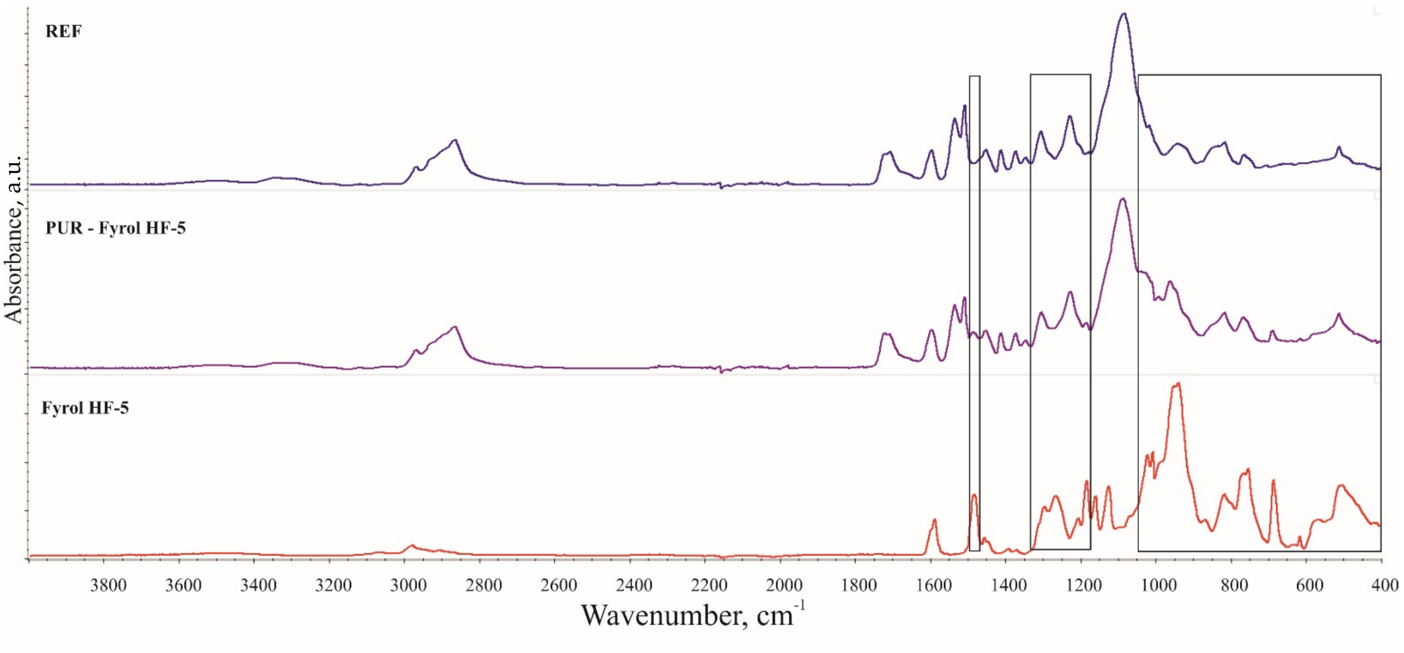

Figure S6. Fourier transform infrared spectroscopy spectra of REF and PUR- Fyrol HF5 foams and the Fyrol HF5 flame retardant.

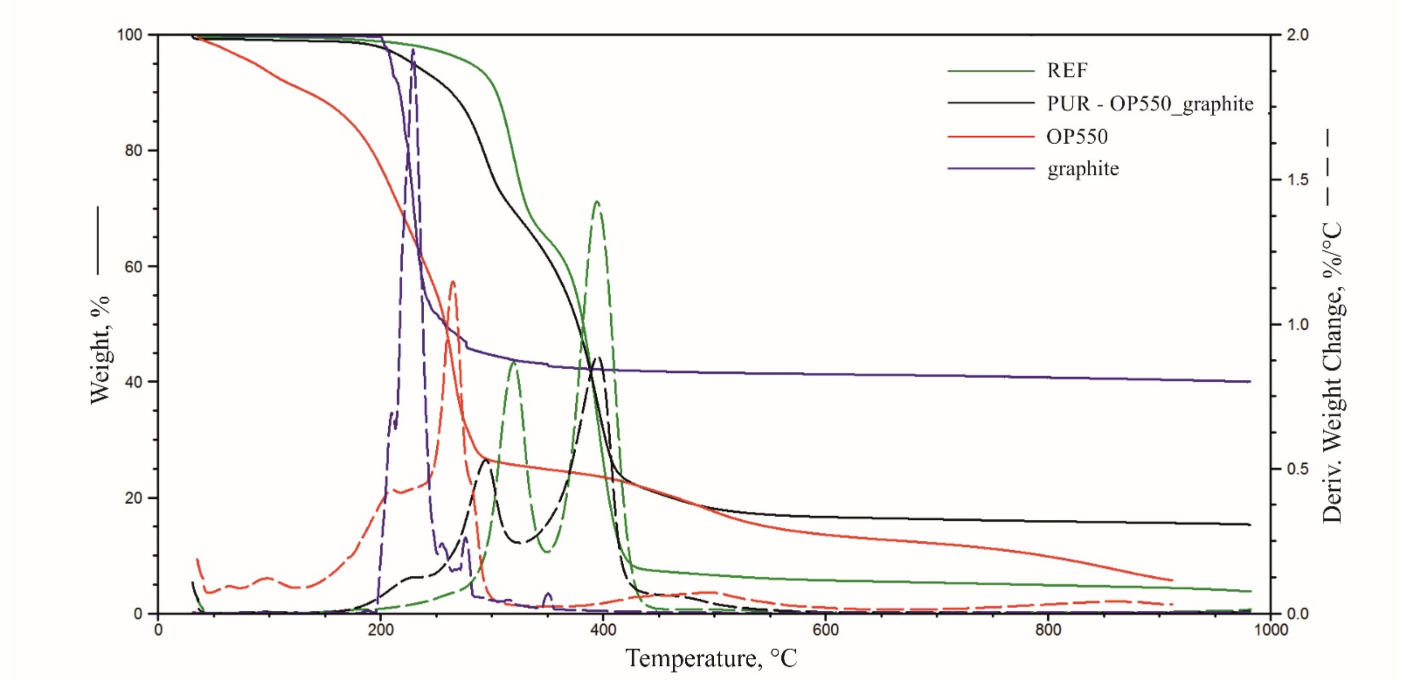

Figure S7. Weight change curves and derivative weight change curves of REF and PUR – OP550_graphite foams and expandable graphite and OP550 flame retardant.

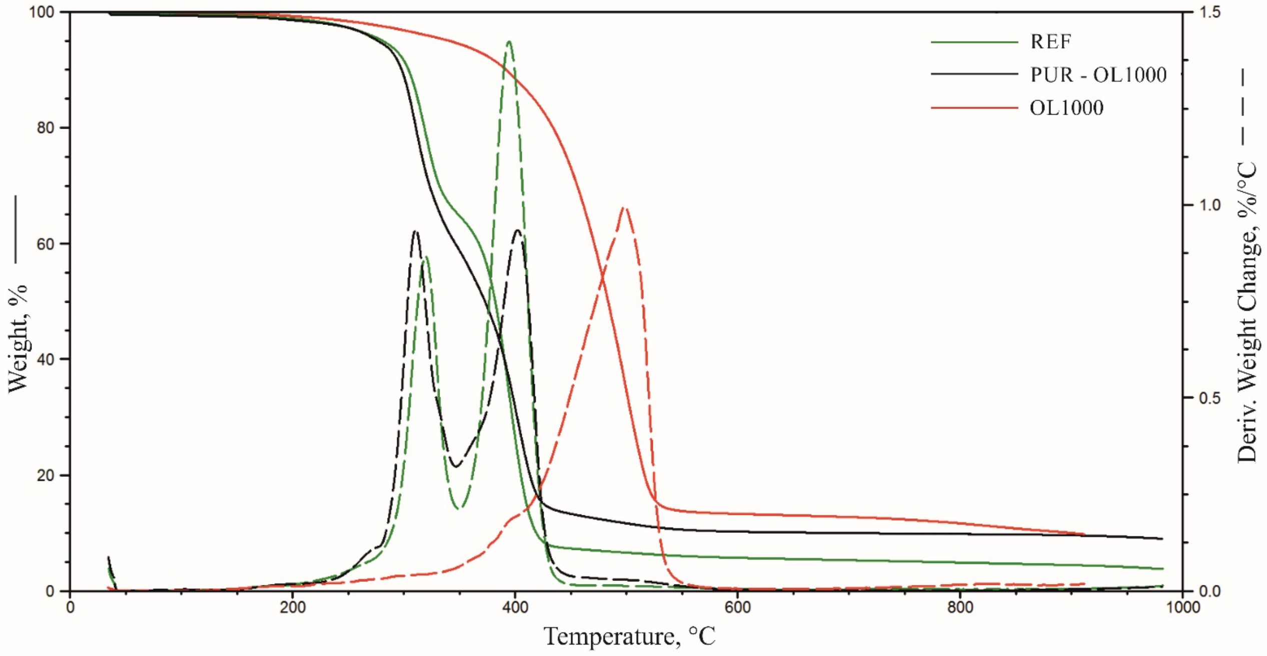

Figure S8. Weight change curves and derivative weight change curves of REF and PUR – OL1000 foams and OL1000 flame retardant.

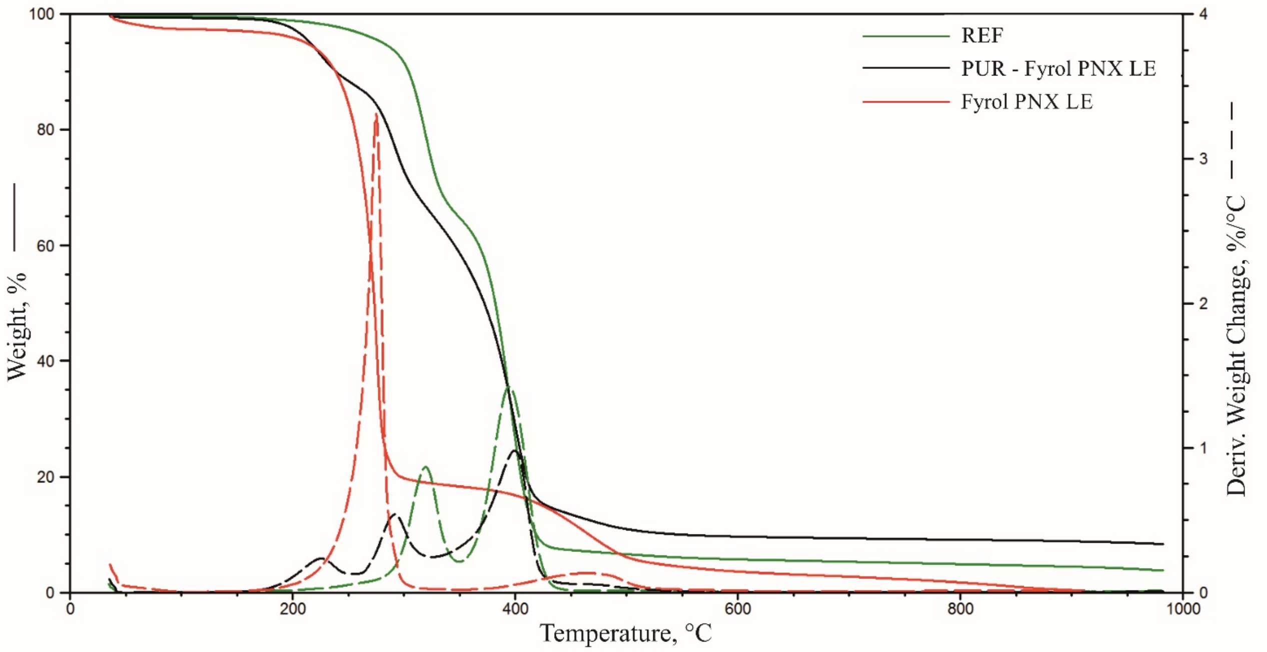

Figure S9. Weight change curves and derivative weight change curves of REF and PUR – Fyrol PNX LE foams and Fyrol PNX LE flame retardant.

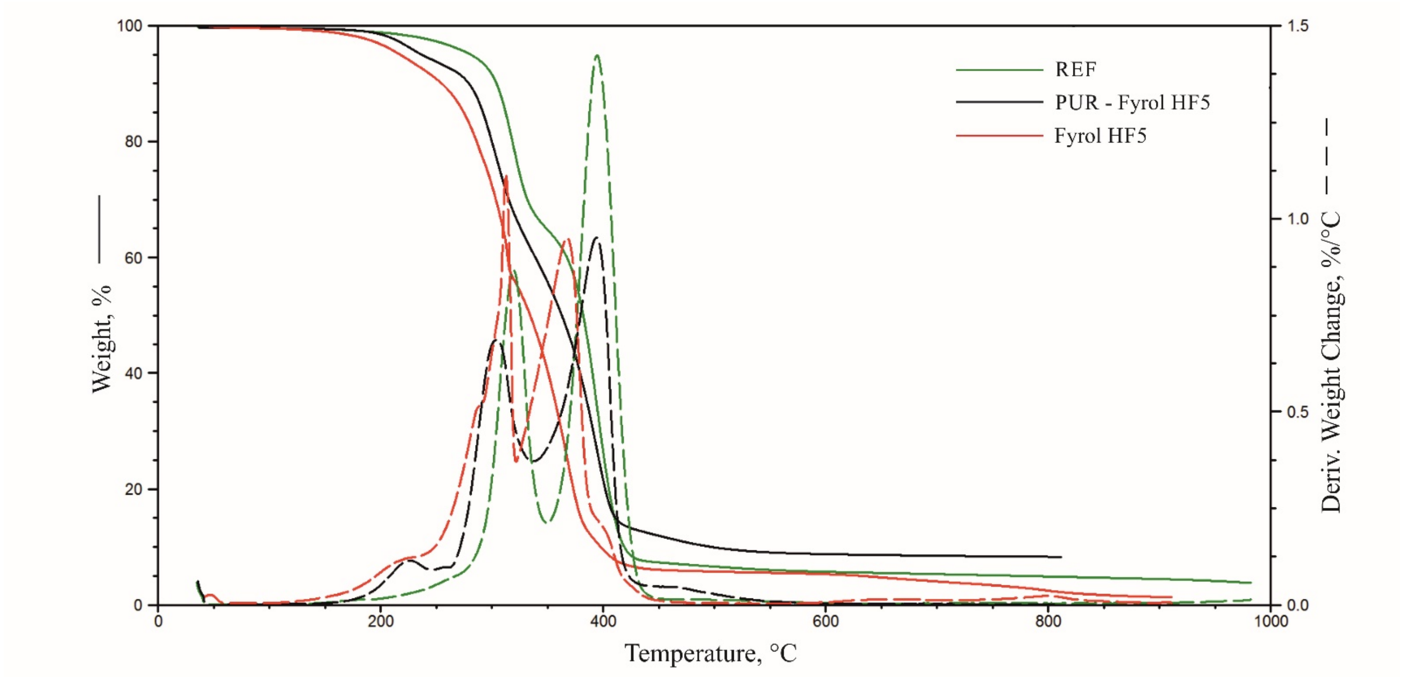

Figure S10. Weight change curves and derivative weight change curves of REF and PUR – Fyrol HF5 foams and Fyrol HF5 flame retardant.

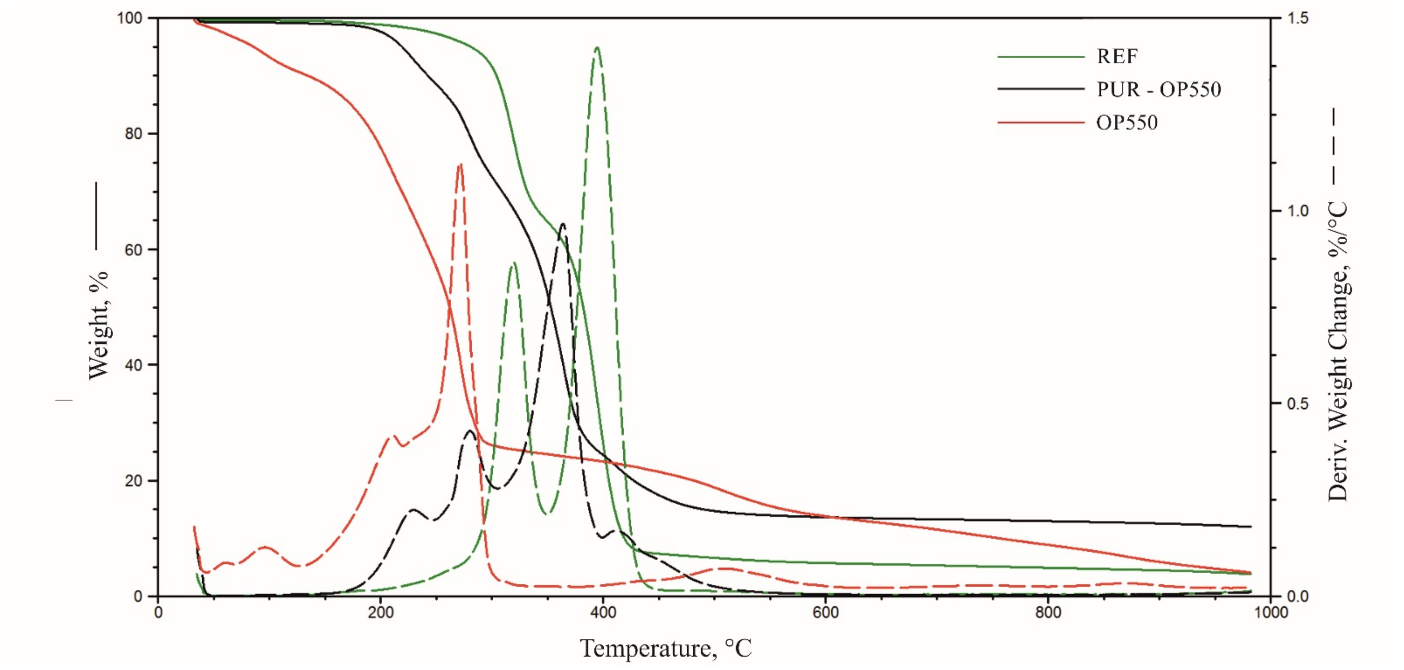

Figure S11. Weight change curves and derivative weight change curves of REF and PUR – OP550 foams and OP550 flame retardant.

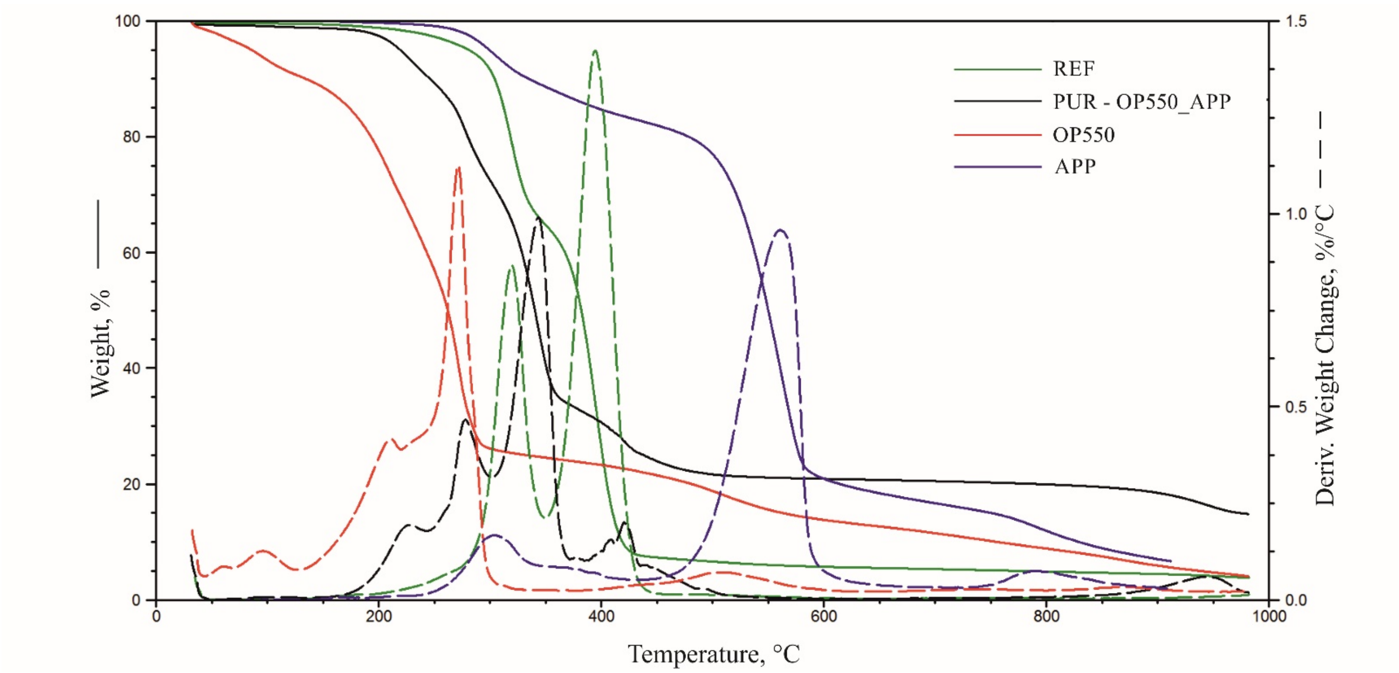

Figure S12. Weight change curves and derivative weight change curves of REF and PUR – OP550_APP foams and OP550 and APP flame retardants.

Table S1. Cytotoxicity of polyurethane foams water extracts towards normal immobilized human keratinocyte cells HaCaT after 24 h exposure as measured in Neutral Red Uptake (NRU) assay. Each value represents the mean of four individual replications from three independent experiments (± SD). Superscript letters mean results significantly different (ANOVA, $p < 0.05$) when comparing the same concentrations.

Foam Type	Concentration (%)	Cytotoxicity (%)
REF	0.78	$-14.30 \pm 0.01^{n' p' r' s'}$
	1.56	$7.55 \pm 0.01^{c' j' k' l'}$
	3.125	15.46 ± 0.02
	6.25	$28.16 \pm 0.01^{s x y z a'}$
	12.5	$34.34 \pm 8.00^{m n o p}$
	25	$66.73 \pm 8.27^{f j k}$
	50	$77.11 \pm 5.46^{a c d e}$
	100	85.13 ± 6.09
PUR–OP 550_graphite	0.78	$-8.73 \pm 0.02^{m' o'}$
	1.56	$-6.36 \pm 0.01^{c' f' j'}$
	3.125	6.10 ± 0.01
	6.25	$0.33 \pm 0.01^{t y}$
	12.5	18.35 ± 11.66
	25	46.75 ± 22.65^{i}
	50	58.10 ± 12.18
	100	72.24 ± 17.40
PUR–OL1000	0.78	$13.36 \pm 0.02^{r'}$
	1.56	$-0.89 \pm 0.02^{c' g'}$
	3.125	14.56 ± 0.03
	6.25	$12.76 \pm 0.03^{t z}$
	12.5	12.16 ± 10.08^{o}
	25	$6.79 \pm 6.45^{g i j}$
	50	$34.57 \pm 10.43^{b c}$

	100	71.10 ± 18.22
PUR–Fyrol PNX LE	0.78	8.42 ± 0.02 $^{s'}$
	1.56	−6.65 ± 0.01
	3.125	9.68 ± 0.01 $^{c' e' h' k'}$
	6.25	7.78 ± 0.01 $^{u x}$
	12.5	30.20 ± 17.59
	25	35.65 ± 26.95 ^{h}
	50	53.53 ± 11.16 ^{d}
	100	76.05 ± 13.52
PUR–Fyrol HF5	0.78	10.73 ± 0.04
	1.56	2.62 ± 0.01 $^{c' i' l'}$
	3.125	12.28 ± 0.02
	6.25	7.73 ± 0.02
	12.5	5.66 ± 6.16 $^{p w a'}$
	25	14.50 ± 9.83
	50	46.10 ± 9.95 ^{e}
	100	75.25 ± 6.60
PUR–OP550	0.78	7.05 ± 0.01 $^{m' n'}$
	1.56	0.83 ± 0.01 $^{d' e'}$
	3.125	−11.24 ± 0.01 $^{b' c'}$
	6.25	1.70 ± 0.01 $^{r s}$
	12.5	0.75 ± 8.76 ^{m}
	25	15.87 ± 14.15 $^{l f}$
	50	44.26 ± 13.19 ^{a}
	100	80.13 ± 18.37
PUR–OP550_APP	0.78	13.46 ± 0.01 $^{o' p'}$
	1.56	22.06 ± 0.04 $^{d' f' g' h' i'}$
	3.125	20.69 ± 0.03 $^{b c}$
	6.25	27.05 ± 0.03 $^{r u w}$
	12.5	10.58 ± 9.62 ^{n}
	25	55.31 ± 6.96 $^{l g h}$
	50	72.93 ± 15.04 ^{b}
	100	79.10 ± 15.62
